# Highly multiplexed immunofluorescence images and single-cell data of immune markers in tonsil and lung cancer

**DOI:** 10.1038/s41597-019-0332-y

**Published:** 2019-12-17

**Authors:** Rumana Rashid, Giorgio Gaglia, Yu-An Chen, Jia-Ren Lin, Ziming Du, Zoltan Maliga, Denis Schapiro, Clarence Yapp, Jeremy Muhlich, Artem Sokolov, Peter Sorger, Sandro Santagata

**Affiliations:** 1 Department of Pathology, Brigham and Women’s Hospital, Harvard Medical School, Boston, MA United States; 2 Laboratory for Systems Pharmacology, Harvard Medical School, Boston, MA United States; 3 Ludwig Center at Harvard, Harvard Medical School, Boston, MA United States; 4 Department of Biomedical Informatics, Harvard Medical School, Boston, MA United States; 5 Broad Institute of MIT and Harvard, Cambridge, MA United States; 6 Department of Systems Biology, Harvard Medical School, Boston, MA United States; 7 Department of Oncologic Pathology, Dana Farber Cancer Institute, Boston, MA United States

**Keywords:** Cancer imaging, Image processing, Diagnostic markers

## Abstract

In this data descriptor, we document a dataset of multiplexed immunofluorescence images and derived single-cell measurements of immune lineage and other markers in formaldehyde-fixed and paraffin-embedded (FFPE) human tonsil and lung cancer tissue. We used tissue cyclic immunofluorescence (t-CyCIF) to generate fluorescence images which we artifact corrected using the BaSiC tool, stitched and registered using the ASHLAR algorithm, and segmented using ilastik software and MATLAB. We extracted single-cell features from these images using HistoCAT software. The resulting dataset can be visualized using image browsers and analyzed using high-dimensional, single-cell methods. This dataset is a valuable resource for biological discovery of the immune system in normal and diseased states as well as for the development of multiplexed image analysis and viewing tools.

## Abstract (summary)

**Table:** 

Measurement(s)	immunofluorescence • biomarker • cellular feature
Technology Type(s)	immunofluorescence microscopy assay • computational modeling technique
Factor Type(s)	Lung carcinoma • Reactive tonsil
Sample Characteristic - Organism	Homo sapiens

Machine-accessible metadata file describing the reported data: 10.6084/m9.figshare.11184539

## Background & Summary

Tissues comprise individual cells of diverse types along with supportive membranes and structures as well as blood and lymphatic vessels. The identities, properties and spatial distributions of cells that make up tissues are still not fully known: classical histology provides excellent spatial resolution, but it typically lacks molecular details. As a result, the impact of intrinsic factors such as lineage and extrinsic factors such as the microenvironment on tissue biology in health and disease requires molecular profiling of single cells within the broader context of organized tissue architecture. Such deep spatial and molecular phenotyping is especially pertinent to the study of cancer resection tissues. These samples are routinely acquired prior to, on, and after a therapeutic intervention, providing opportunities to characterize the interplay between malignant tumor cells and surrounding immune cell populations and how those relationships are influenced over time by treatments. Understanding these relationships may elucidate biomarker signatures that predict response to therapy^1,2^ and is particularly relevant in the case of immunotherapeutics. Many available immunotherapies, including those targeting cytotoxic T lymphocyte-associated antigen-4 (CTLA-4), programmed cell death-1 receptor (PD-1), and programmed cell death-1 ligand (PD-L1), influence interactions between tumor and immune cells to inhibit immune checkpoints and activate the immune system’s surveillance of tumor cells^3–7^. However, even in tumor types that are highly responsive to such therapies, many patients do not benefit, and many types of tumors remain broadly refractory to these agents. A deeper understanding of immune cell states, location, interactions, and architecture (“immunophenotypes”) promises to provide new prognostic and predictive information for cancer research and treatment.

With recent advances in multiplexed imaging technologies^8^, multiple epitopes can be detected within a tissue section and the spatial distributions and interactions of cell populations precisely mapped. One such method is tissue-based cyclic immunofluorescence (t-CyCIF)^9^ which yields high-plex images at subcellular resolution and has been used to characterize immune populations in several tumor types^10–13^. In t-CyCIF, a high-plex image is constructed from a series of 4 to 6 color images, which are then registered and superimposed. The images provide information on the amount of epitope that is expressed as well as the location of the epitope within the tissue. By segmenting the images to demarcate single cells or subcellular compartments, we can then use epitope expression levels to discriminate immune, tumor, and stromal cell types and compute their numbers and distributions within tumors and surrounding normal tissue.

The quality of the antibody reagents largely dictates the reliability of data that is generated by antibody-based imaging methods such as multiplexed ion beam imaging (MIBI)^14^, imaging mass cytometry (IMC)^15^, co-detection by indexing (CODEX)^16^, DNA exchange imaging (DEI)^17^, MultiOmyx (MxIF)^18^, imaging cycler microscopy (ICM)^19–21^, multiplexed IHC^22^, NanoString Digital Spatial Profiling (DSP)^23^, and t-CyCIF itself. We have recently published detailed methods for validating antibodies and assembling panels of antibodies for multiplexed tissue techniques^24^. That work highlights a variety of complementary approaches to qualify antibodies using information at the level of pixels, cells, and tissues and yielded a 16-plex antibody panel capable of detecting lymphocytes, macrophages, and immune checkpoint regulators for use in ‘immune profiling’ tissue samples. Using t-CyCIF, we qualified antibodies in reactive (non-neoplastic) tonsil tissue (TONSIL-1), which has a highly stereotyped arrangement of diverse immune cell types, and then demonstrated the panel’s utility in characterizing common and rare immune populations in three lung cancer tissue specimens: a lung adenocarcinoma that had metastasized to a lymph node (LUNG-1-LN), a lung squamous cell carcinoma that had metastasized to the brain (LUNG-2-BR), and a primary lung squamous cell carcinoma (LUNG-3-PR). We also provide t-CyCIF imaging data from eight FFPE sections used to validate antibodies; in these samples, antibodies were applied in different permutations and order, making the data useful for examining relationships between antigenicity, fluorescence signal, and cycle number.

In this data descriptor, we share the images from our recent work^24^. The dataset includes immunofluorescence images from formalin fixed paraffin embedded (FFPE) tissue sections mounted onto glass slides. In each section, there are between ~61,800 to ~483,000 individual cells with fluorescence intensity and spatial information provided for 27 antibodies that were acquired in a multiplexed fashion. These antibodies include the highly validated 16-plex immune panel as well as antibodies against several additional markers of interest such as markers of tumor cell lineage and cell proliferation. We also include quantitative, single-cell measurements of 60+ features including fluorescence intensity measurements for each target epitope/protein, cellular morphology measurements such as area, eccentricity, and solidity, and spatial information such as the centroid position of each cell and its nearest neighbors.

The resulting single-cell data can be analyzed using qualitative and quantitative approaches both in the context of the original spatial arrangement of the tissue and as sets of derived feature vectors, one for each cell. Spatial views enable the analysis of geographic patterns and interactions between different cells types, such as the immune microenvironment surrounding tumor tissue. Such data can be used to develop new methods for visualizing large complex images and to develop and refine data analysis approaches such as image segmentation, intensity gating (to discriminate ‘positive’ and ‘negative’ cell populations), and spatial clustering. As multiple research centers begin to assemble high-dimensional and multi-parametric atlases of human cancers and pre-cancers^25^, there is an increasing need for cross-center validation of analysis methodologies. Publicly available datasets such as ours will provide a freely accessible resource for such efforts.

## Methods

### Tissue samples

Five formalin-fixed paraffin-embedded (FFPE) human tissue samples were retrieved from the archives of the Department of Pathology at Brigham and Women’s Hospital with IRB approval as part of a discarded tissue protocol. The diagnoses were confirmed by a board-certified pathologist (S.S.) (Table 1). Sections were cut from FFPE blocks at a thickness of 5 µm and mounted onto Superfrost Plus microscope slides prior to use.

**Table 1:** Sample Information.

Sample Code	Data Set	Tissue Type	Clinical Classification
TONSIL-1	1	Human tonsil tissue	Normal tonsil
LUNG-1-LN	1	Human lung carcinoma tissue	Lung adenocarcinoma metastasis to lymph node
LUNG-2-BR	1	Human lung carcinoma tissue	Lung squamous cell carcinoma metastasis to brain
LUNG-3-PR	1	Human lung carcinoma tissue	Primary lung squamous cell carcinoma
TONSIL-2.1	2	Human tonsil tissue	Reactive tonsil
TONSIL-2.2	2	Human tonsil tissue	Reactive tonsil
TONSIL-2.3	2	Human tonsil tissue	Reactive tonsil
TONSIL-2.4	2	Human tonsil tissue	Reactive tonsil
TONSIL-2.5	2	Human tonsil tissue	Reactive tonsil
TONSIL-2.6	2	Human tonsil tissue	Reactive tonsil
TONSIL-2.7	2	Human tonsil tissue	Reactive tonsil
TONSIL-2.8	2	Human tonsil tissue	Reactive tonsil

### Datasets

Data from tissue samples was acquired in two batches. The first batch (DATASET-1) contains data from LUNG-1-LN, LUNG-2-BR, LUNG-3-PR, and TONSIL-1. The second batch (DATASET-2) contains data from eight sections of TONSIL-2. Data associated with each of these sections are labeled TONSIL-2.1, TONSIL-2.2, etc. in the data records. Note that in the sample coding system, the number after the dash denotes patient sample and the number after the decimal point denotes block section.

### Tissue-based cyclic immunofluorescence

**Fig. 1:**
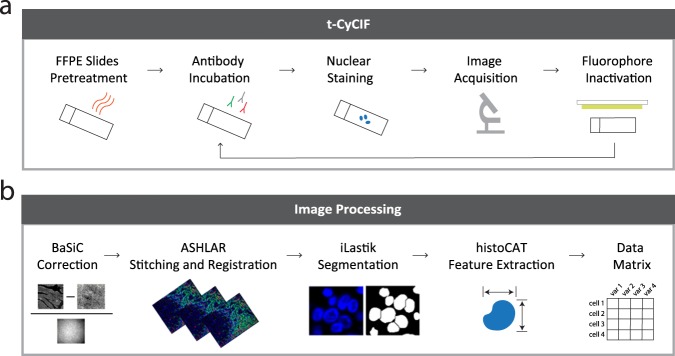
Overview of data generation. (**a**) Multiplexed, immunofluorescence images were acquired using the tissue-based cyclic immunofluorescence (t-CyCIF) method and (**b**) processed with a series of algorithms and toolboxes including BaSiC, ASHLAR, ilastik, and histoCAT to obtain single-cell features.

Each section of tissue was imaged with a panel of 26–28 antibodies using t-CyCIF as previously described^9^. This method consists of iterative cycles of antibody incubation, imaging, and fluorophore inactivation (Fig. 1).

#### Slide preparation

An automated program on the Leica Bond RX (Leica Biosystems) was used to prepare slides for t-CyCIF. The slides were treated as follows: baked at 60 °C for 30 min, dewaxed at 72 °C with Bond Dewax Solution (Cat. AR9222, Leica Biosystems), and treated with Epitope Retrieval 1 (ER1) Solution at 100 °C for 20 min for antigen retrieval. Odyssey Blocking Buffer (Cat. 927–40150, LI-COR) was applied to the slides at room temperature (RT) for 30 min and then incubated with three secondary antibodies at RT for 60 min, followed by Hoechst 33342 (Cat. H3570, Life Technologies) solution (2 ug/ml) at RT for 30 min.

#### Blocking

After slide preparation, non-specific, reactive epitopes were blocked by incubating slides overnight at 4 °C in the dark with fluorescently conjugated secondary antibodies raised against the host species of the unconjugated, primary antibodies used in the first cycle of t-CyCIF.

#### Antibody staining

Slides were initially imaged to measure nonspecific binding from secondary antibodies, photobleached, and then imaged again to measure tissue autofluorescence. In the first cycle of antibody incubation, the slides were incubated overnight with primary antibodies from different species and then with corresponding secondary antibodies for two hours at RT in the dark. Slides were then washed with 1X PBS, stained with Hoechst solution, and then imaged. This process was repeated for 11–12 cycles using antibodies directly conjugated to fluorophores. All antibodies used in this study are listed in Online-only Table 1 with an assigned unique identifier. Antibodies and imaging parameters used for each cycle of imaging for all samples in DATASET-1 are detailed in Online-only Table 2 and for all samples in DATASET-2 in Online-only Table 3.

#### Mounting and de-coverslipping

Prior to each cycle of imaging, slides were wet-mounted using 200 µl of 10% glycerol in PBS and 24 × 50 mm glass cover slips (Cat # 48393-081, VWR). Following imaging, the slides were de-coverslipped by placing the slides vertically in a slide rack completely submerged in a container of 1X PBS for 15 minutes and slowly pulling the slides back up, allowing the glass coverslip to remain in the PBS.

#### Image acquisition

Images from each cycle of t-CyCIF were acquired using the RareCyte CyteFinder Slide Scanning Fluorescence Microscope. The four following filter sets were used: 1) The ‘DAPI channel’ for imaging Hoechst with a peak excitation of 390 nm and half-width of 18 nm and a peak emission of 435 nm and half-width of 48 nm, 2) the ‘488 channel’ with a 475-nm/28-nm excitation filter and a 525-nm/48-nm emission filter, 3) the ‘555 channel’ with a 542-nm/27-nm excitation filter and a 597-nm/45-nm emission filter, and 4) the ‘647 channel’ with a 632-nm/22-nm excitation filter and a 679-nm/34-nm emission filter. Each tissue section was imaged twice, a large region with a 10X/0.3 NA objective and a smaller region with a 40X/0.6NA objective. The 10X images have a field of view of 1.6 × 1.4 mm and a nominal resolution of 1.06 µm. The 40X images have a field of view of 0.42 × 0.35 mm and a nominal resolution of 0.53 µm. For both sets of images, a 5% overlap was collected between fields of view to facilitate image stitching. In DATASET-2, the first cycle of antibodies was imaged twice, once with a high exposure time and once with a low exposure time.

#### Photobleaching

Following slide preparation using the Leica Bond RX and subsequent to each cycle of imaging, fluorophores were inactivated by submerging slides in a solution of 4.5% H_2_O_2_ and 20 mM NaOH in 1X PBS and incubating them under a light emitting diode (LED) for 2 hours at RT.

### Image processing

#### Background and shading correction

The BaSiC algorithm^26^ plugin for ImageJ was used to computationally derive flat-field and dark-field profiles from the original image for each cycle. The flat-field is used to correct for irregular illumination of the sample, and the dark-field is used to correct for camera sensor offset and internal noise. Lambda values of 0.1 and 0.01 were used for flat-field and dark-field, respectively. For each cycle, the raw image was subtracted by the dark-field profile and divided by the flat-field profile to correct the shading on each individual image field.

#### Stitching and registration

ASHLAR (version v1.6.0) was used to stitch the fields from the first imaging cycle into a mosaic and to co-register the fields from successive cycles of imaging. Ashlar stitches fields together by calculating the phase correlation between neighboring images to correct for local state positioning error and applying a statistical model of microscope stage behavior to correct for large-scale error. It then uses a similar phase correlation approach to register fields from successive cycles to the first cycle of stitched images. The output is an OME-TIFF file that contains a seamless multi-channel mosaic depicting the entire sample across all image cycles.

#### Segmentation

The OME-TIFF output from ASHLAR was used to segment single cells in the images using the ilastik software program^27^ and MATLAB (version 2018a). The OME-TIFF was cropped into 6000 × 6000 pixel regions to increase processing speed. From each cropped region, ~20 random 250 × 250 pixel regions were selected and used as training data in the ilastik program to generate a probability of each pixel in the cropped region belonging to three classes: nuclear area, cytoplasmic area, or area not occupied by a cell (background). During the labeling process, the user was presented with the DAPI channel only. The user labeled pixels with DAPI as nuclei, pixels on the border or a few pixels away from DAPI signal as cytoplasm, and pixels distant from DAPI signal as background. While labeling by the user was performed using only one DAPI channel, all 44 channels from the stitched and registered images were used by ilastik to train the pixel classification algorithm. Color/intensity features including gaussian smoothing, edge features including the Laplacian of gaussian, gaussian of gradient magnitude, and difference of gaussians, and texture features including structure tensor eigenvalues and hessian of gaussian eigenvalues with a σ_0_ = 0.30, σ_1_ = 0.70, σ_2_ = 1.00, σ_3_ = 1.60, σ_4_ = 3.50, and σ_5_ = 5.03 were used to train the pixel classification in ilastik. The ilastik software generated three probability masks, one for each of the three classes. For example, the cytoplasmic probability mask was a TIFF image, with each pixel containing a value between 0 to 65535 where larger values indicate higher probability of that pixel belonging to the cytoplasmic class. The probability masks along with morphological manipulations were used in MATLAB to perform a watershed transformation and identify objects, or cell nuclei. The output from MATLAB was a nuclear segmentation mask for each cropped region. Please see below for a description of the qualitative and quantitative approaches we used for the technical validation and assessment of the segmentation.

#### Single-cell feature extraction

The histology topography cytometry analysis toolbox (histoCAT)^28^ was used to extract features of the cells segmented in each image. Single cell features included fluorescence intensity measurements of each antibody, morphological features such as cell area and circularity, as well as spatial features such as the centroid position of the cell. Moreover, cells in spatial proximity to one another were identified and indexed to enable neighborhood analysis and cell phenotype interactions. The output was a data table for each cropped region. For each sample, the data tables from all the cropped regions were concatenated into a master image level data table with each cell assigned a global unique identifier and centroid position. A complete list and description of each feature in the master data tables is provided in Online-only Table 4.

## Data Records

**Fig. 2:**
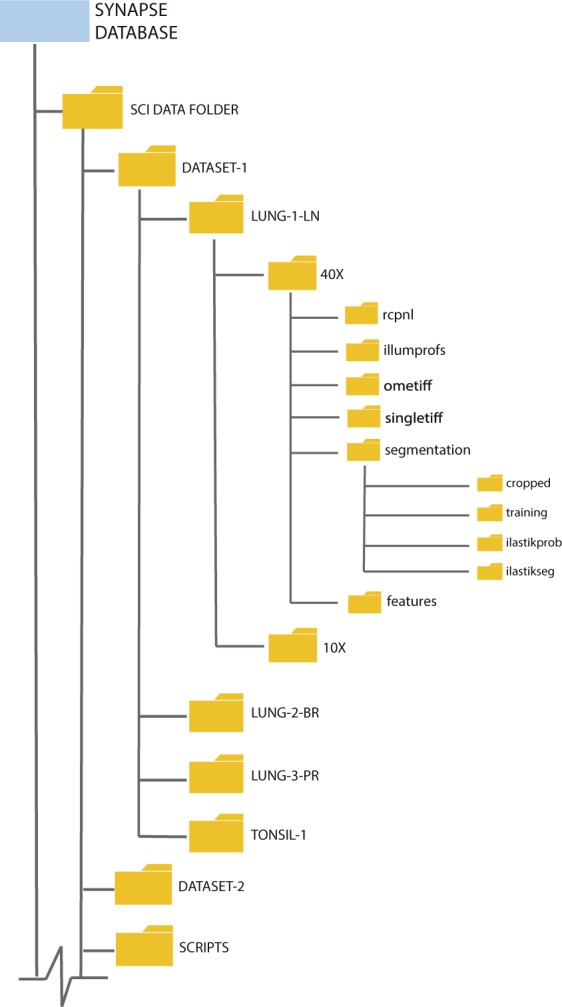
Database structure. All shared data are stored in the SYNAPSE repository. 10.7303/syn17865732

We have made all the data for this manuscript available in the Synapse repository hosted by Sage Bionetworks 10.7303/syn17865732 ^29^. We organized the data as described in Fig. 2. For each tissue sample, we share image data acquired at two magnifications. For each 40X magnification in DATASET-1, we share:

i. raw rcpnl files,
ii. illumination profiles generated by the BaSiC algorithm,
iii. an OME-TIFF file output from the ASHLAR algorithm,
iv. individual TIFF images for each marker,
v. probability masks for segmentation from ilastik software,
vi. labeled nuclear segmentation mask, and
vii. data table of 60+ features extracted for each cell.

The “rcpnl” folder contains the raw image files in an rcpnl file format generated by the RareCyte CyteFinder for each cycle of imaging. The “illumprofs” folder contains TIFF files for the dark-field profile and the flat-field profile for each cycle of imaging. Each TIFF file in this folder is a stack of four TIFF images corresponding to the four wavelengths imaged every cycle. The “ometiff” folder contains one OME-TIFF file that is a stitched, registered mosaic of all channels across all cycles of imaging. The OME-TIFF file has a pyramidal structure that contains mosaics at multiple resolutions. The “singletiff” folder contains a single TIFF mosaic for each marker at the highest resolution. This folder separates the OME-TIFF into separate channels to facilitate opening in software that is incompatible with the OME-TIFF format. The “segmentation” folder contains subfolders with intermediate data outputs from the segmentation process. The “cropped” subfolder contains 6000 × 6000 pixel regions from the OME-TIFF file. The “training” subfolder contains 250 × 250 pixel regions used as training data for segmentation. The “ilastikprob” subfolder contains a TIFF image for the probability of each pixel in the cropped regions belonging to each class used in ilastik training. The “ilastikseg” folder contains a TIFF image of the nuclear segmentation mask. This folder also contains an TIFF image stack with the segmentation mask and the DAPI fluorescence image from the first cycle of imaging for easy comparison of the accuracy of the probability mask. The “features” folder contains a csv data table for each cropped region with 60+ feature measurements for each cell as well as a master data table with data from each cropped region combined. Note that the X and Y coordinates for the centroid of the cell in the master table reflects the global position of the cell in the entire piece of tissue imaged/stitched image.

We provide all scripts used in data generation. A description of the scripts and supporting documents is provided in Online-only Table 5.

Additionally, a subset of the imaging data can be found and viewed on cycif.org (https://www.cycif.org/featured-paper/du-lin-rashid-2019/figures/). In this interactive image browser, we indicate several distinct regions of interest in the tonsil and lung cancer images and provide descriptive narrations about a subset of the combinations of immune markers expressed in these samples.

## Technical Validation

### Staining quality

**Fig. 3:**
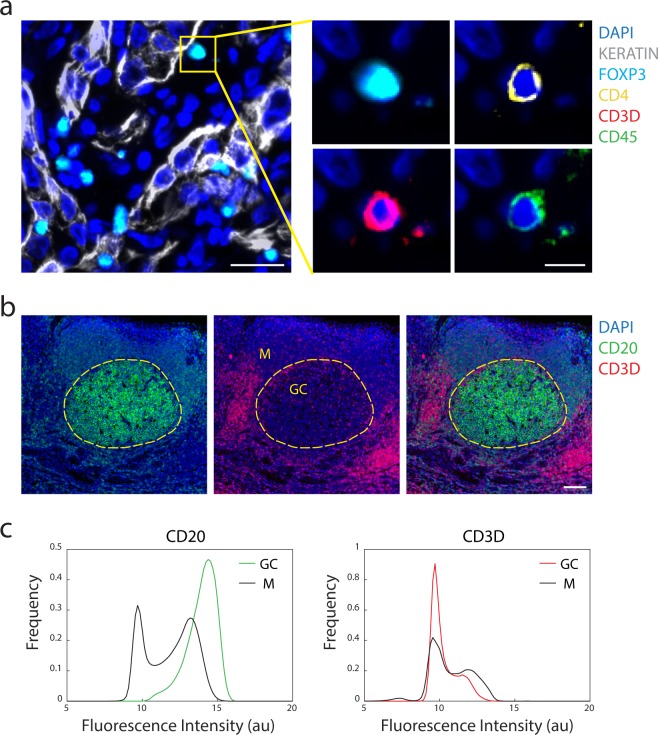
Antibody staining quality. (**a**) Immunofluorescence image from LUNG-3-PR showing epithelial tumor cells marked by Keratin (white) and a regulatory T cell marked by FOXP3 (cyan), CD4 (yellow), CD3D (red), and CD45 (green) (scale bar: 25 µm; inset scale bar: 10 µm). (**b**) A region of TONSIL-1 showing CD20 (green) and CD3D (red) expression. Area inside yellow dashed circle denotes germinal center (GC), and area outside denotes the mantle (M) region (scale bar: 100 µm). (**c**) Probability density function of fluorescence signal intensity of every pixel in the germinal center (n = 1,446,450 pixels) and mantle (n = 4,369,358 pixels) for CD20 and CD3D within the region shown in (**b**). X-axis is fluorescence intensity (log2 au) and y-axis is frequency of pixels.

We performed a detailed validation of the panel of antibodies used to generate the datasets described in our prior work^24^. One or more trained pathologists visually reviewed the staining patterns for each antibody to assess specificity to cell type, appropriate localization within the cell (e.g. nucleus v. cytoplasm v. membrane), co-staining with other markers, and localization to the expected geographic regions within the tissue. For example, the cytokeratin antibody, known to detect intermediate filament proteins in epithelial cells, was expressed in striated patterns surrounding the nuclei of cells morphologically consistent with epithelial origin, whereas the FOXP3 antibody, targeting a transcription factor in T cells, was concentrated in the nuclear area of small, round cells morphologically consistent with lymphocytes (Fig. 3a). Antibodies detecting cell lineage markers such as FOXP3, which delineates a regulatory T-cell population, were further corroborated by assessing appropriate co-expression of other markers. For example, we found that FOXP3 was co-expressed with CD4, CD3D, and CD45, thereby increasing our confidence in the staining quality (Fig. 3a). As another example, CD20, a B-cell antigen, was observed to have higher levels of signal within germinal centers of tonsil tissue which are well-established B cell rich compartments within tonsil rather than the mantle region where we found an abundance of cells expressing the T-cell antigen CD3D (Fig. 3b). See our prior publication^23^ for additional quality measurements including the comparison of t-CyCIF antibody staining to the staining observed with clinical grade antibodies that were used in immunohistochemistry (IHC) staining, pixel-by-pixel correlations of multiple antibody clones against the same target, and various high-dimensional cell clustering methods.

### Cell segmentation

**Fig. 4:**
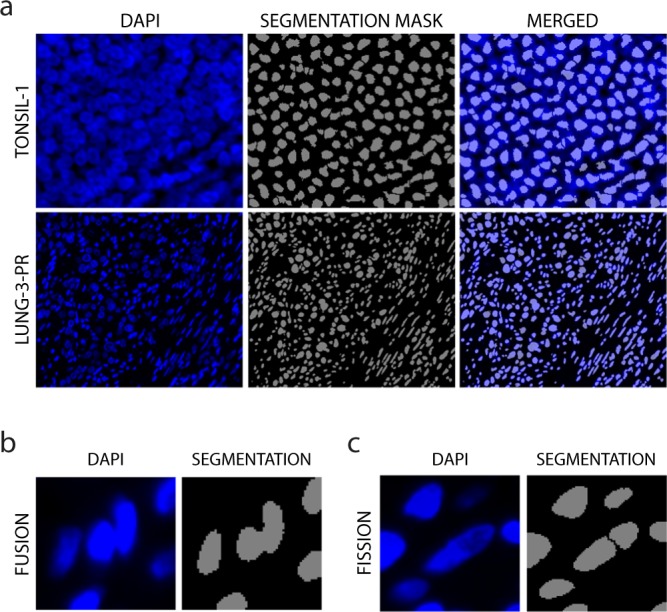
Assessment of segmentation. (**a**) Representative images of DAPI staining and corresponding segmentation mask in TONSIL-1 and LUNG-3-PR. (**b**) Examples of fusion (under-segmentation) and (**c**) fission/splitting (over-segmentation).

We evaluated the quality of segmentation of single cells within the tissue images using a two-step system. We only performed segmentation on the 40X magnification images because the lower resolution of the 10X magnification images reduced segmentation accuracy. First, we overlaid the segmentation masks over the DAPI signal to evaluate the accuracy of segmentation qualitatively (Fig. 4a); based on these data, we then adjusted and optimized the segmentation. Second, three users evaluated a random sample of 500 cells from the tonsil and each of the lung tissues to quantify the accuracy, or true positives, and rate of fusion errors (under-segmentation) and fission/splitting errors (over-segmentation) among mis-segmented cells (Fig. 4b-c, Table 2). The cell segmentation of all samples had a low error rate (~0.1) across cells of various morphologies (large tumor cells, smaller round immune cells, elongated fibroblasts, etc.). The accuracy of image segmentation can be further improved with the development of new algorithms.

**Table 2:** Segmentation Accuracy.

	LUNG-1-LN		LUNG-2-BR		LUNG-3-PR		TONSIL-1	
	Mean	SD	Mean	SD	Mean	SD	Mean	SD
True Positive	0.872	0.013	0.890	0.017	0.899	0.013	0.863	0.012
Fusion	0.086	0.027	0.074	0.017	0.053	0.019	0.038	0.020
Fission	0.042	0.017	0.036	0.003	0.047	0.008	0.099	0.025

In our analysis of these images, we observed that the area covered by the nuclear mask effectively captured the signal from the nuclear compartment as well as the cytoplasmic/membranous compartment as can be observed in Fig. 3a. The presence of cytoplasmic signal in the nuclear compartment in this dataset is in part attributable to the three-dimensional nature of the five-micron thick tissue sections which we imaged. These sections capture the complex intermingling of nuclear and cytoplasmic compartments that occurs in individual cells. Thus, the signal that is ultimately projected into a two-dimensional image does not arise strictly from one cellular compartment. Moreover, the high cellular density in these tumor and tonsil tissues in combination with high intensity fluorescence signal created conditions where expanding the nuclear segmentation mask captured signal from neighboring cells. Therefore, in our single-cell analyses, we used the nuclear segmentation mask to extract signal intensity features for both nuclear and cytoplasmic markers.

### Single-cell feature extraction

**Fig. 5:**
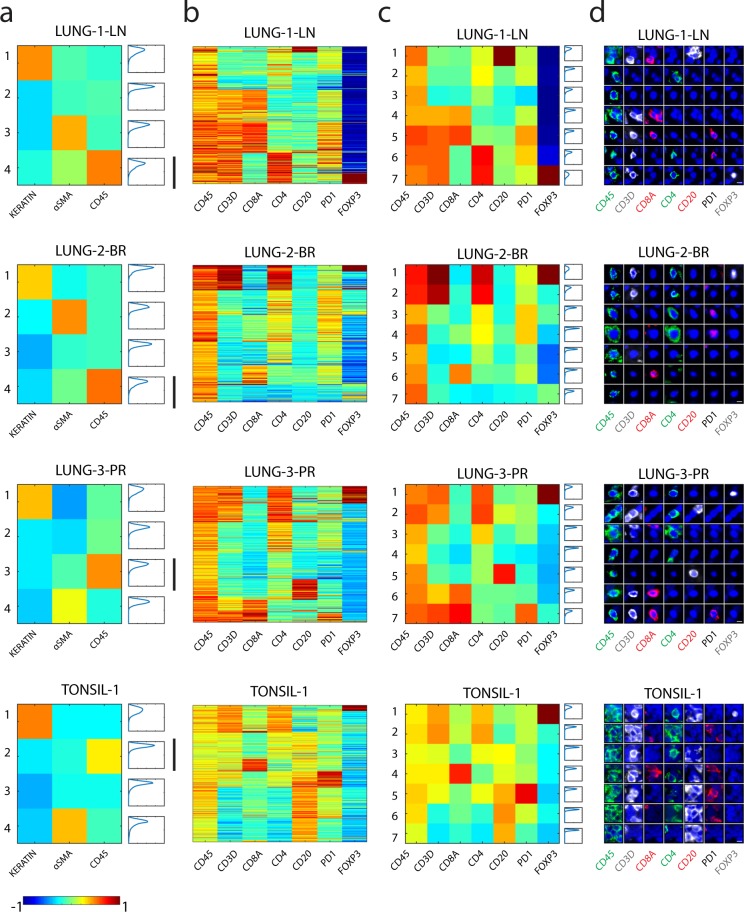
Heatmaps of cell populations from lung cancer and tonsil tissues using k-means clustering demonstrates distinct cell immune populations with expected patterns of biomarker expression. (**a**) Heatmap of the expression of Keratin, αSMA, and CD45 in all cells that were collected from LUNG-1-LN, LUNG-2-BR, LUNG-3-PR, and TONSIL-1 using k-means clustering. Each row is a cluster. The last column in each heat map shows the probability density function (pdf) plot showing the fit of each cell within the cluster, with the x-axis denoting the frequency of cells and y-axis denoting the Euclidean distance of the cell from the centroid of the cluster. The black vertical bars mark the immune cluster with high CD45 expression. (**b**,**c**) Heatmaps showing the expression of seven lymphocyte markers (CD45, CD3D, CD8A, CD4, CD20, PD1, FOXP3) from the cells within the CD45 high cluster from panel (a). (**b**) Each row represents protein marker expression data from a single cell or (**c**) each row represents a cluster. Note that fluorescence intensity values were log transformed and normalized between –1 to 1 as indicated by the color bar. (**d**) Galleries of immunofluorescence images of representative cells from each cluster in (**c**). (Scale bar: 5 µm).

To assess the integrity of the single-cell features extracted from the images, we applied an unsupervised, k-means clustering method to the data from the three lung cancer resection samples and the reactive (non-neoplastic) tonsil sample. This analysis yielded four cardinal cell types (clusters) using three lineage markers (Fig. 5a). For each sample, the cells clustered into an epithelial group marked by keratin expression, a stromal group marked by αSMA expression, and an immune group marked by CD45 expression. A fourth group was marked by low expression of all three markers. We then isolated the cells in the immune group and further clustered them using other lymphocyte markers (Fig. 5b,c). The clustering revealed similar immune cell populations to those observed by visual review of the images and as quantified previously using other computational methods^24^. Each cluster exhibited varying degrees of tightness, or fit. The probability density function plot for each cluster in Fig. 5a,c displays the distance of each cell from the centroid of the cluster, with the y-axis denoting distance and the x-axis denoting the frequency of cells belonging to each distance bin. The range of the curve along the y-axis reflects the fit, with a smaller range denoting greater fit and a larger range denoting poorer fit. The variability of cluster fit can be explained by the intrinsic heterogeneity within different immune populations. Tighter clusters where the majority of the cells have short distances from the center represent populations with distinct and highly similar marker expression profiles. Looser clusters, with wider distance ranges and longer tails, likely contain subpopulations of immune cells that may require further stratification and investigation. While this exercise displays fundamental immune cell populations reported in the literature, we note the potential of multiplexed data and unsupervised methods to reveal novel cell populations and states. Here, using alternative segmentation, feature extraction, and computational approaches, we retained reproducible immune cell populations, giving us confidence in the robustness of this dataset.

## Usage Notes

More information on the t-CyCIF method used to generate this data can be found at: www.cycif.org and a detailed protocol can be found in Lin *et al*.^9^ and Du, Lin, Rashid *et al*. 2019^24^.

A narrative of the dataset is available for interactive web-browsing here: https://www.cycif.org/featured-paper/du-lin-rashid-2019/figures/.

Open data agreement: Highly multiplexed immunofluorescence images and single-cell data of immune markers in tonsil and lung cancer by Rumana Rashid, Giorgio Gaglia, Yu-An Chen, Jia-Ren Lin, Ziming Du, Zoltan Maliga, Denis Schapiro, Clarence Yapp, Jeremy Muhlich, Artem Sokolov, Peter Sorger and Sandro Santagata is licensed under a Creative Commons Attribution-ShareAlike 4.0 International License. Based on a work at 10.7303/syn17865732.

## Data Availability

All code used to process and generate the data in this study can be found alongside the dataset^29^. A description of each script is provided in Online-only Table 5. Source code for ASHLAR is available on GitHub (https://github.com/jmuhlich/ashlar). The newest histoCAT version can also be found GitHub (https://github.com/BodenmillerGroup/histoCAT).

## Online-only Tables

**Online-only Table 1:** Antibody Unique Identifiers.

ID	Name	Vendor	Catalog
1	CD68	Cell Signaling Technology	79594
2	CD3	BioLegend	300422
3	CD11a	BioLegend	301207
4	CD15	BioLegend	301910
5	CD16	BioLegend	302019
6	CD19	BioLegend	302219
7	CD25	BioLegend	302617
8	CD28	BioLegend	302954
9	CD38	BioLegend	303511
10	CD45	BioLegend	304056
11	CD64	BioLegend	305012
12	CD80	BioLegend	305207
13	CD83	BioLegend	305308
14	CD86	BioLegend	305405
15	CD86	BioLegend	305416
16	CD123	BioLegend	306035
17	CD69	BioLegend	310904
18	CD206	BioLegend	321116
19	EpCam	BioLegend	324205
20	Her2	BioLegend	324412
21	CD1c	BioLegend	331505
22	CD305	BioLegend	342802
23	CD134	BioLegend	350018
24	CD103	BioLegend	350209
25	Ki67	BioLegend	350509
26	CD138	BioLegend	352308
27	TIM1	BioLegend	353904
28	CD25	BioLegend	356104
29	CD27	BioLegend	356406
30	CD49b	BioLegend	359305
31	CD33	BioLegend	366608
32	ABCC1	BioLegend	370203
33	IFNG	BioLegend	502517
34	CD16	BD Biosiences	558122
35	GATA3	BDBiosiences	560163
36	pH2AX	BioLegend	613412
37	Annexin V	BioLegend	640911
38	NFATc1	BioLegend	649605
39	Beta-catenin	BioLegend	658705
40	VIM	BioLegend	677807
41	CD11a	eBioscience	11-0119-41
42	Ki67	Cell Signaling Technology	11882 s
43	CD66b	Thermo-Fisher	12-0666-41
44	Ki67	Cell Signaling Technology	12075 S
45	CD133	eBioscience	12-1338-41
46	VEGFR2	Cell Signaling Technology	12634 S
47	pAur	Cell Signaling Technology	13464 S
48	STING	Cell Signaling Technology	13647 S
49	IRF1	Cell Signaling Technology	14105 S
50	PD-L1	Cell Signaling Technology	15005 S
51	Beta-Tubulin	Cell Signaling Technology	2116 S
52	pH3	Cell Signaling Technology	3475 S
53	CD45R	Invitrogen	41-0452-80
54	CD4	eBioscience	41-2444-82
55	FoxP3	eBioscience	41-4777-82
56	Keratin	eBioscience	41-9003-82
57	Her2	eBioscience	41-9757-80
58	CD11c	eBioscience	41-9761-80
59	Vinculin	eBioscience	41-9777-80
60	GFAP	eBioscience	41-9892-80
61	CD11c	Cell Signaling Technology	45581 S
62	CD8a	eBioscience	50-0008-82
63	CD3	eBioscience	50-0037-41
64	CD20	eBioscience	50-0202-80
65	aSMA	eBioscience	50-9760-82
66	RunX3	eBioscience	50-9817-80
67	CD11b	eBioscience	53-0196-80
68	CD45RB	eBioscience	53-9458-80
69	TIM3	Cell Signaling Technology	54669 S
70	EGFR	Cell Signaling Technology	5616 S
71	PDL2	Cell Signaling Technology	82723 S
72	PCNA	Cell Signaling Technology	8580 S
73	LaminA/C	Cell Signaling Technology	8617 S
74	Axl	Cell Signaling Technology	8661 S
75	CD1c	Abcam	ab156708
76	LAG3	Abcam	ab180187
77	CD115	Abcam	ab183316
78	LaminA/C	Abcam	ab185014
79	g Tubulin	Abcam	ab191114
80	TDP43	Abcam	ab193842
81	Lamin B	Abcam	ab194108
82	IBA1	Abcam	ab195031
83	CD14	Abcam	ab196169
84	CD19	Abcam	ab196468
85	Fibronectin	Abcam	ab198933
86	STING	Abcam	ab198952
87	HLA-A	Abcam	ab199837
88	CD1a	Abcam	ab201337
89	PD-1	Abcam	ab201825
90	aSMA	Abcam	ab202509
91	CD21	Abcam	ab202693
92	CD69	Abcam	ab202909
93	SQSTM1	Abcam	ab203430
94	CD11b	Abcam	ab204271
95	FOXO1A	Abcam	ab207244
96	S100a	Abcam	ab207367
97	CD3	Abcam	ab208514
98	BANF1	Abcam	ab208534
99	CTLA4	Abcam	ab210254
100	PML	Abcam	ab217524
101	CD163	Abcam	ab218293
102	PKR	Abcam	ab219739
103	CD2	Abcam	ab37212
104	IBA1	Bioss	AIF1
105	BRD7	Aviva	ARP39018-P050
106	CD45	R&D Systems	FAB1430P-025
107	CD31	R&D Systems	FAB3567P
108	CD4	R&D Systems	FAB8165G
109	CD45RO	Dako	M0742
110	GATA3	Thermo-Fisher	MA1-028
111	IDO	EMD-Millipore	MAB10009
112	RORyT	EMD-Millipore	MABF81
113	CCR7	Invitrogen	PA5-32299
114	CD16	Santa Cruz	sc-20052 AF647
115	CD209	Santa Cruz	sc-65740
116	p-cJun	Santa Cruz	sc-822
117	Arl13b	Antibodies Inc.	75-287
118	CD45RO	Dako	M0742
119	Hoechst 33342	Cell Signaling Technology	4082 S

**Online-only Table 2:** Antibody Staining Plan for DATASET-1.

channel_number	cycle_number	marker_name	fluorescence_label	wavelength_name	excitation_wavelength	emission_wavelength	antibody_ID	antibody_vendor	antibody_catalog	antibody_dilution	exposure_time (sec)
1	1	DAPI_1	Hoechst 33342	DAPI	395	431	119	Cell Signaling Technology	4082 S	1:1000	0.1
2	1	A488 background		FITC	485	525					1
3	1	A555 background		Cy3	555	590					1
4	1	A647 background		Cy5	640	690					1
5	2	DAPI_2	Hoechst 33342	DAPI	395	431	119	Cell Signaling Technology	4082 S	1:1000	0.05
6	2	A488 background	Alexa 488	FITC	485	525					1
7	2	A555 background	Alexa 555	Cy3	555	590					1
8	2	A647 background	Alexa 647	Cy5	640	690					1
9	3	DAPI_3	Hoechst 33342	DAPI	395	431	119	Cell Signaling Technology	4082 S	1:1000	0.075
10	3	A488 background	Alexa 488	FITC	485	525					1
11	3	LAG3	Alexa 555	Cy3	555	590	76	Abcam	ab180187	1:100	1
12	3	ARL13B	Alexa 647	Cy5	640	690	117	Antibodies Incorporated	75-287	1:100	1
13	4	DAPI_4	Hoechst 33342	DAPI	395	431	119	Cell Signaling Technology	4082 S	1:1000	0.05
14	4	KI67	Alexa 488	FITC	485	525	42	Cell Signaling Technology	11882 s	1:100	1
15	4	KERATIN	Alexa 555	Cy3	555	590	56	eBioscience	41-9003-80	1:200	1
16	4	PD1	Alexa 647	Cy5	640	690	89	Abcam	ab201825	1:100	1
17	5	DAPI_5	Hoechst 33342	DAPI	395	431	119	Cell Signaling Technology	4082 S	1:1000	0.02
18	5	CD45RB	Alexa 488	FITC	485	525	68	eBioscience	53-9458-80	1:100	1
19	5	CD3D	Alexa 555	Cy3	555	590	97	Abcam	ab208514	1:100	1
20	5	PDL1	Alexa 647	Cy5	640	690	50	Cell Signaling Technology	15005 S	1:50	1
21	6	DAPI_6	Hoechst 33342	DAPI	395	431	119	Cell Signaling Technology	4082 S	1:1000	0.02
22	6	CD4	Alexa 488	FITC	485	525	108	R&D Systems	FAB8165G	1:100	1
23	6	CD45	Alexa 555	Cy3	555	590	106	R&D Systems	FAB1430P-025	1:100	1
24	6	CD8A	Alexa 647	Cy5	640	690	62	eBioscience	50-0008-80	1:100	1
25	7	DAPI_7	Hoechst 33342	DAPI	395	431	119	Cell Signaling Technology	4082 S	1:1000	0.05
26	7	CD163	Alexa 488	FITC	485	525	101	Abcam	ab218293	1:100	1
27	7	CD68	Alexa 555	Cy3	555	590	1	Cell Signaling Technology	79594	1:100	1
28	7	CD14	Alexa 647	Cy5	640	690	83	Abcam	ab196169	1:100	0.75
29	8	DAPI_8	Hoechst 33342	DAPI	395	431	119	Cell Signaling Technology	4082 S	1:1000	0.15
30	8	CD11B	Alexa 488	FITC	485	525	67	eBioscience	53-0196-80	1:100	0.75
31	8	FOXP3	Alexa 555	Cy3	555	590	55	eBioscience	41-4777-80	1:100	1
32	8	CD21	Alexa 647	Cy5	640	690	91	Abcam	ab202693	1:100	0.2
33	9	DAPI_9	Hoechst 33342	DAPI	395	431	119	Cell Signaling Technology	4082 S	1:1000	0.05
34	9	IBA1	Alexa 488	FITC	485	525	82	Abcam	ab195031	1:250	0.75
35	9	ASMA	Alexa 555	Cy3	555	590	90	Abcam	ab202509	1:250	0.2
36	9	CD20	Alexa 647	Cy5	640	690	64	eBioscience	50-0202-80	1:250	0.2
37	10	DAPI_10	Hoechst 33342	DAPI	395	431	119	Cell Signaling Technology	4082 S	1:1000	0.1
38	10	CD19	Alexa 488	FITC	485	525	94	Abcam	ab196468	1:100	1
39	10	GFAP	Alexa 555	Cy3	555	590	60	eBioscience	41-9892-80	1:100	0.1
40	10	GTUBULIN	Alexa 647	Cy5	640	690	79	Abcam	ab191114	1:100	1
41	11	DAPI_11	Hoechst 33342	DAPI	395	431	119	Cell Signaling Technology	4082 S	1:1000	0.075
42	11	LAMINAC	Alexa 488	FITC	485	525	78	Abcam	ab185014	1:100	0.5
43	11	BANF1	Alexa 555	Cy3	555	590	98	Abcam	ab208534	1:100	1
44	11	LAMINB	Alexa 647	Cy5	640	690	81	Abcam	ab194108	1:100	0.4

**Online-only Table 3:** Antibody Staining Plan for DATASET-2.

sample	channel_number	cycle_number	marker_name	fluorescence_label	wavelength_name	excitation_wavelength	emission_wavelength	antibody_ID	antibody_vendor	antibody_catalog	anitbody_dilution	exposure_time(sec)
TONSIL-2.1	1	1	DAPI_1	Hoechst 33342	DAPI	395	431	119	Cell Signaling Technology	4082 S	1:1000	0.03
TONSIL-2.1	2	1	A488 background		FITC	485	525					0.5
TONSIL-2.1	3	1	A555 background		Cy3	555	590					0.5
TONSIL-2.1	4	1	A647 background		Cy5	640	690					0.5
TONSIL-2.1	5	2	DAPI_2	Hoechst 33342	DAPI	395	431	119	Cell Signaling Technology	4082 S	1:1000	0.03
TONSIL-2.1	6	2	A488 background		FITC	485	525					0.2
TONSIL-2.1	7	2	A555 background		Cy3	555	590					0.2
TONSIL-2.1	8	2	A647 background		Cy5	640	690					0.2
TONSIL-2.1	9	3	DAPI_3	Hoechst 33342	DAPI	395	431	119	Cell Signaling Technology	4082 S	1:1000	0.03
TONSIL-2.1	10	3	CD11c	Alexa 488	FITC	485	525	61	Cell Signaling Technology	45581 S	1:300	0.5
TONSIL-2.1	11	3	A555 background		Cy3	555	590					0.5
TONSIL-2.1	12	3	CD209	Alexa 647	Cy5	640	690	115	Santa Cruz	sc-65740	1:100	0.5
TONSIL-2.1	13	4	DAPI_4	Hoechst 33342	DAPI	395	431	119	Cell Signaling Technology	4082 S	1:1000	0.03
TONSIL-2.1	14	4	CD11c	Alexa 488	FITC	485	525	61	Cell Signaling Technology	45581 S	1:300	0.2
TONSIL-2.1	15	4	A555 background		Cy3	555	590					0.2
TONSIL-2.1	16	4	CD209	Alexa 647	Cy5	640	690	115	Santa Cruz	sc-65740	1:100	0.2
TONSIL-2.1	17	5	DAPI_5	Hoechst 33342	DAPI	395	431	119	Cell Signaling Technology	4082 S	1:1000	0.03
TONSIL-2.1	18	5	CD4	Alexa 488	FITC	485	525	108	R&D Systems	FAB8165G	1:300	0.5
TONSIL-2.1	19	5	CD68	Alexa 555	Cy3	555	590	1	Cell Signaling Technology	79594 S	1:1000	0.5
TONSIL-2.1	20	5	CD20	Alexa 647	Cy5	640	690	64	eBioscience	50-0202-80	1:1000	0.5
TONSIL-2.1	21	6	DAPI_6	Hoechst 33342	DAPI	395	431	119	Cell Signaling Technology	4082 S	1:1000	0.03
TONSIL-2.1	22	6	PCNA	Alexa 488	FITC	485	525	72	Cell Signaling Technology	8580 S	1:1000	0.2
TONSIL-2.1	23	6	CD4	Alexa 555	Cy3	555	590	54	eBioscience	41-2444-82	1:200	0.5
TONSIL-2.1	24	6	CD14	Alexa 647	Cy5	640	690	83	Abcam	ab196169	1:1000	0.2
TONSIL-2.1	25	7	DAPI_7	Hoechst 33342	DAPI	395	431	119	Cell Signaling Technology	4082 S	1:1000	0.03
TONSIL-2.1	26	7	EGFR	Alexa 488	FITC	485	525	70	Cell Signaling Technology	5616 S	1:500	0.5
TONSIL-2.1	27	7	CD11c	Alexa 555	Cy3	555	590	58	eBioscience	41-9761-80	1:3'1:3'1:3	0.5
TONSIL-2.1	28	7	VIM	Alexa 647	Cy5	640	690	40	BioLegend	677807	1:500	0.1
TONSIL-2.1	29	8	DAPI_8	Hoechst 33342	DAPI	395	431	119	Cell Signaling Technology	4082 S	1:1000	0.03
TONSIL-2.1	30	8	IBA1	Alexa 488	FITC	485	525	82	Abcam	ab195031	1:500	0.5
TONSIL-2.1	31	8	CD86	Alexa 555	Cy3	555	590	14	BioLegend	305405	1:100	0.5
TONSIL-2.1	32	8	CD45	Alexa 647	Cy5	640	690	10	BioLegend	304056	1:300	0.5
TONSIL-2.1	33	9	DAPI_9	Hoechst 33342	DAPI	395	431	119	Cell Signaling Technology	4082 S	1:1000	0.03
TONSIL-2.1	34	9	CD11b	Alexa 488	FITC	485	525	94	Abcam	ab204271	1:300	0.5
TONSIL-2.1	35	9	CD3D	Alexa 555	Cy3	555	590	97	Abcam	ab208514	1:1'1:50	0.5
TONSIL-2.1	36	9	CD64	Alexa 647	Cy5	640	690	11	BioLegend	305012	1:1'1:50	0.5
TONSIL-2.1	37	10	DAPI_10	Hoechst 33342	DAPI	395	431	119	Cell Signaling Technology	4082 S	1:1000	0.03
TONSIL-2.1	38	10	CD19	Alexa 488	FITC	485	525	6	BioLegend	302219	1:200	0.5
TONSIL-2.1	39	10	FoxP3	Alexa 555	Cy3	555	590	55	eBioscience	41-4777-82	1:1'1:50	0.5
TONSIL-2.1	40	10	CD134	Alexa 647	Cy5	640	690	23	BioLegend	350018	1:300	0.5
TONSIL-2.1	41	11	DAPI_11	Hoechst 33342	DAPI	395	431	119	Cell Signaling Technology	4082 S	1:1000	0.03
TONSIL-2.1	42	11	IFNG	Alexa 488	FITC	485	525	33	BioLegend	502517	1:1'1:50	0.5
TONSIL-2.1	43	11	PML	Alexa 555	Cy3	555	590	100	Abcam	ab217524	1:1'1:50	0.5
TONSIL-2.1	44	11	CD305	Alexa 647	Cy5	640	690	22	BioLegend	342802	1:1'1:50	0.5
TONSIL-2.1	45	12	DAPI_12	Hoechst 33342	DAPI	395	431	119	Cell Signaling Technology	4082 S	1:1000	0.03
TONSIL-2.1	46	12	TIm3	Alexa 488	FITC	485	525	69	Cell Signaling Technology	54669 S	1:200	0.5
TONSIL-2.1	47	12	Keratin	Alexa 555	Cy3	555	590	56	eBioscience	41-9003-82	1:1000	0.1
TONSIL-2.1	48	12	CD8a	Alexa 647	Cy5	640	690	62	eBioscience	50-0008-82	1:200	0.5
TONSIL-2.2	1	1	DAPI_1	Hoechst 33342	DAPI	395	431	119	Cell Signaling Technology	4082 S	1:1000	0.03
TONSIL-2.2	2	1	A488 background		FITC	485	525					0.5
TONSIL-2.2	3	1	A555 background		Cy3	555	590					0.5
TONSIL-2.2	4	1	A647 background		Cy5	640	690					0.5
TONSIL-2.2	5	2	DAPI_2	Hoechst 33342	DAPI	395	431	119	Cell Signaling Technology	4082 S	1:1000	0.03
TONSIL-2.2	6	2	A488 background		FITC	485	525					0.2
TONSIL-2.2	7	2	A555 background		Cy3	555	590					0.2
TONSIL-2.2	8	2	A647 background		Cy5	640	690					0.2
TONSIL-2.2	9	3	DAPI_3	Hoechst 33342	DAPI	395	431	119	Cell Signaling Technology	4082 S	1:1000	0.03
TONSIL-2.2	10	3	CCR7	Alexa 488	FITC	485	525	113	Invitrogen	PA5-32299	1:100	0.5
TONSIL-2.2	11	3	A555 background		Cy3	555	590					0.5
TONSIL-2.2	12	3	CD45RO	Alexa 647	Cy5	640	690	118	Dako	M0742	1:300	0.5
TONSIL-2.2	13	4	DAPI_4	Hoechst 33342	DAPI	395	431	119	Cell Signaling Technology	4082 S	1:1000	0.03
TONSIL-2.2	14	4	CCR7	Alexa 488	FITC	485	525	113	Invitrogen	PA5-32299	1:100	0.2
TONSIL-2.2	15	4	A555 background		Cy3	555	590					0.2
TONSIL-2.2	16	4	CD45RO	Alexa 647	Cy5	640	690	1118	Dako	M0742	1:300	0.2
TONSIL-2.2	17	5	DAPI_5	Hoechst 33342	DAPI	395	431	119	Cell Signaling Technology	4082 S	1:1000	0.03
TONSIL-2.2	18	5	CD11b	Alexa 488	FITC	485	525	94	Abcam	ab204271	1:300	0.5
TONSIL-2.2	19	5	CD3D	Alexa 555	Cy3	555	590	97	Abcam	ab208514	1:1'1:50	0.5
TONSIL-2.2	20	5	CD16	PacBlue	Cy5	640	690	34	BD Biosiences	558122	1:1'1:50	0.5
TONSIL-2.2	21	6	DAPI_6	Hoechst 33342	DAPI	395	431	119	Cell Signaling Technology	4082 S	1:1000	0.03
TONSIL-2.2	22	6	GATA3	Alexa 488	FITC	485	525	35	BD Biosiences	560163	1:100	0.5
TONSIL-2.2	23	6	EpCam	Alexa 555	Cy3	555	590	19	BioLegend	324205	1:300	0.5
TONSIL-2.2	24	6	CD45	Alexa 647	Cy5	640	690	10	BioLegend	304056	1:300	0.5
TONSIL-2.2	25	7	DAPI_7	Hoechst 33342	DAPI	395	431	119	Cell Signaling Technology	4082 S	1:1000	0.03
TONSIL-2.2	26	7	CD4	Alexa 488	FITC	485	525	108	R&D Systems	FAB8165G	1:500	0.5
TONSIL-2.2	27	7	FoxP3	Alexa 555	Cy3	555	590	55	eBioscience	41-4777-82	1:2'1:50	0.5
TONSIL-2.2	28	7	CD20	Alexa 647	Cy5	640	690	64	eBioscience	50-0202-80	1:1'1:6'1:70	0.2
TONSIL-2.2	29	8	DAPI_8	Hoechst 33342	DAPI	395	431	119	Cell Signaling Technology	4082 S	1:1000	0.03
TONSIL-2.2	30	8	CD69	Alexa 488	FITC	485	525	17	BioLegend	310904	1:100	0.5
TONSIL-2.2	31	8	Keratin	Alexa 555	Cy3	555	590	56	eBioscience	41-9003-82	1:1000	0.1
TONSIL-2.2	32	8	CD8a	Alexa 647	Cy5	640	690	62	eBioscience	50-0008-82	1:200	0.5
TONSIL-2.2	33	9	DAPI_9	Hoechst 33342	DAPI	395	431	119	Cell Signaling Technology	4082 S	1:1000	0.03
TONSIL-2.2	34	9	IBA	Alexa 488	FITC	485	525	82	Abcam	ab195031	1:500	0.5
TONSIL-2.2	35	9	CD25	PE	Cy3	555	590	28	BioLegend	356104	1:1'1:50	0.5
TONSIL-2.2	36	9	CD86	Alexa 647	Cy5	640	690	15	BioLegend	305416	1:1'1:50	0.5
TONSIL-2.2	37	10	DAPI_10	Hoechst 33342	DAPI	395	431	119	Cell Signaling Technology	4082 S	1:1000	0.03
TONSIL-2.2	38	10	PCNA	Alexa 488	FITC	485	525	72	Cell Signaling Technology	8580 S	1:1000	0.2
TONSIL-2.2	39	10	CD45R	Alexa 555	Cy3	555	590	53	Invitrogen	41-0452-80	1:200	0.5
TONSIL-2.2	40	10	CD14	Alexa 647	Cy5	640	690	83	Abcam	ab196169	1:1000	0.5
TONSIL-2.2	41	11	DAPI_11	Hoechst 33342	DAPI	395	431	119	Cell Signaling Technology	4082 S	1:1000	0.03
TONSIL-2.2	42	11	CD15	Alexa 488	FITC	485	525	4	BioLegend	301910	1:300	0.2
TONSIL-2.2	43	11	CD27	PE	Cy3	555	590	29	BioLegend	356406	1:1'1:50	0.2
TONSIL-2.2	44	11	PDL1	Alexa 647	Cy5	640	690	50	Cell Signaling Technology	15005 S	1:300	0.5
TONSIL-2.2	45	12	DAPI_12	Hoechst 33342	DAPI	395	431	119	Cell Signaling Technology	4082 S	1:1000	0.03
TONSIL-2.2	46	12	CD163	Alexa 488	FITC	485	525	101	Abcam	ab218293	1:300	0.5
TONSIL-2.2	47	12	CD68	Alexa 555	Cy3	555	590	1	Cell Signaling Technology	79594 S	1:1000	0.5
TONSIL-2.2	48	12	HLA-A	Alexa 647	Cy5	640	690	87	Abcam	ab199837	1:200	0.2
TONSIL-2.3	1	1	DAPI_1	Hoechst 33342	DAPI	395	431	119	Cell Signaling Technology	4082 S	1:1000	0.03
TONSIL-2.3	2	1	A488 background		FITC	485	525					0.5
TONSIL-2.3	3	1	A555 background		Cy3	555	590					0.5
TONSIL-2.3	4	1	A647 background		Cy5	640	690					0.5
TONSIL-2.3	5	2	DAPI_2	Hoechst 33342	DAPI	395	431	119	Cell Signaling Technology	4082 S	1:1000	0.03
TONSIL-2.3	6	2	A488 background		FITC	485	525					0.2
TONSIL-2.3	7	2	A555 background		Cy3	555	590					0.2
TONSIL-2.3	8	2	A647 background		Cy5	640	690					0.2
TONSIL-2.3	9	3	DAPI_3	Hoechst 33342	DAPI	395	431	119	Cell Signaling Technology	4082 S	1:1000	0.03
TONSIL-2.3	10	3	CD115	Alexa 488	FITC	485	525	77	Abcam	ab183316	1:100	0.5
TONSIL-2.3	11	3	A555 background		Cy3	555	590					0.5
TONSIL-2.3	12	3	CD1a	Alexa 555	Cy5	640	690	88	Abcam	ab201337	1:30	0.5
TONSIL-2.3	13	4	DAPI_4	Hoechst 33342	DAPI	395	431	119	Cell Signaling Technology	4082 S	1:1000	0.03
TONSIL-2.3	14	4	CD115	Alexa 488	FITC	485	525	77	Abcam	ab183316	1:100	0.2
TONSIL-2.3	15	4	A555 background		Cy3	555	590					0.2
TONSIL-2.3	16	4	CD1a	Alexa 647	Cy5	640	690	88	Abcam	ab201337	1:30	0.2
TONSIL-2.3	17	5	DAPI_5	Hoechst 33342	DAPI	395	431	119	Cell Signaling Technology	4082 S	1:1000	0.03
TONSIL-2.3	18	5	PCNA	Alexa 488	FITC	485	525	72	Cell Signaling Technology	8580 S	1:1000	0.5
TONSIL-2.3	19	5	FoxP3	Alexa 555	Cy3	555	590	55	eBioscience	41-4777-82	1:1'1:50	0.5
TONSIL-2.3	20	5	IRF1	Alexa 647	Cy5	640	690	49	Cell Signaling Technology	14105 S	1:200	0.5
TONSIL-2.3	21	6	DAPI_6	Hoechst 33342	DAPI	395	431	119	Cell Signaling Technology	4082 S	1:1000	0.03
TONSIL-2.3	22	6	Lamin A/C	Alexa 488	FITC	485	525	73	Cell Signaling Technology	8617 S	1:300	0.5
TONSIL-2.3	23	6	Keratin	Alexa 555	Cy3	555	590	56	eBioscience	41-9003-82	1:1000	0.1
TONSIL-2.3	24	6	CD3	Alexa 647	Cy5	640	690	63	eBioscience	50-0037-41	1:300	0.5
TONSIL-2.3	25	7	DAPI_7	Hoechst 33342	DAPI	395	431	119	Cell Signaling Technology	4082 S	1:1000	0.03
TONSIL-2.3	26	7	CD11b	Alexa 488	FITC	485	525	94	Abcam	ab204271	1:500	0.5
TONSIL-2.3	27	7	Vinculin	Alexa 555	Cy3	555	590	59	eBioscience	41-9777-80	1:500	0.5
TONSIL-2.3	28	7	CD14	Alexa 647	Cy5	640	690	83	Abcam	ab196169	1:1'1:6'1:70	0.2
TONSIL-2.3	29	8	DAPI_8	Hoechst 33342	DAPI	395	431	119	Cell Signaling Technology	4082 S	1:1000	0.03
TONSIL-2.3	30	8	CD49b	Alexa 488	FITC	485	525	30	BioLegend	359305	1:100	0.5
TONSIL-2.3	31	8	CD68	Alexa 555	Cy3	555	590	1	Cell Signaling Technology	79594 S	1:1000	0.5
TONSIL-2.3	32	8	IBA1	Alexa 647	Cy5	640	690	104	Bioss	AIF1	1:100	0.5
TONSIL-2.3	33	9	DAPI_9	Hoechst 33342	DAPI	395	431	119	Cell Signaling Technology	4082 S	1:1000	0.03
TONSIL-2.3	34	9	TIM3	Alexa 488	FITC	485	525	69	Cell Signaling Technology	54669 S	1:200	0.5
TONSIL-2.3	35	9	CD33	PE	Cy3	555	590	31	BioLegend	366608	1:1'1:50	0.5
TONSIL-2.3	36	9	CD8a	Alexa 647	Cy5	640	690	62	eBioscience	50-0008-82	1:200	0.5
TONSIL-2.3	37	10	DAPI_10	Hoechst 33342	DAPI	395	431	119	Cell Signaling Technology	4082 S	1:1000	0.03
TONSIL-2.3	38	10	CD28	Alexa 488	FITC	485	525	8	BioLegend	302954	1:1'1:50	0.5
TONSIL-2.3	39	10	CD3D	Alexa 555	Cy3	555	590	97	Abcam	ab208514	1:1'1:50	0.5
TONSIL-2.3	40	10	CD45	Alexa 647	Cy5	640	690	10	BioLegend	304056	1:300	0.5
TONSIL-2.3	41	11	DAPI_11	Hoechst 33342	DAPI	395	431	119	Cell Signaling Technology	4082 S	1:1000	0.03
TONSIL-2.3	42	11	PKR-	Alexa 488	FITC	485	525	102	Abcam	ab219739	1:1'1:50	0.5
TONSIL-2.3	43	11	CD83	PE	Cy3	555	590	13	BioLegend	305308	1:1'1:50	0.5
TONSIL-2.3	44	11	CD206	Alexa 647	Cy5	640	690	18	BioLegend	321116	1:100	0.5
TONSIL-2.3	45	12	DAPI_12	Hoechst 33342	DAPI	395	431	119	Cell Signaling Technology	4082 S	1:1000	0.03
TONSIL-2.3	46	12	CD4	Alexa 488	FITC	485	525	108	R&D Systems	FAB8165G	1:300	0.5
TONSIL-2.3	47	12	CTLA4	PE	Cy3	555	590	99	Abcam	ab210254	1:200	0.5
TONSIL-2.3	48	12	CD16	APC	Cy5	640	690	34	BD Biosiences	558122	1:200	0.5
TONSIL-2.4	1	1	DAPI_1	Hoechst 33342	DAPI	395	431	119	Cell Signaling Technology	4082 S	1:1000	0.03
TONSIL-2.4	2	1	A488 background		FITC	485	525					0.5
TONSIL-2.4	3	1	A555 background		Cy3	555	590					0.5
TONSIL-2.4	4	1	A647 background		Cy5	640	690					0.5
TONSIL-2.4	5	2	DAPI_2	Hoechst 33342	DAPI	395	431	119	Cell Signaling Technology	4082 S	1:1000	0.03
TONSIL-2.4	6	2	A488 background		FITC	485	525					0.2
TONSIL-2.4	7	2	A555 background		Cy3	555	590					0.2
TONSIL-2.4	8	2	A647 background		Cy5	640	690					0.2
TONSIL-2.4	9	3	DAPI_3	Hoechst 33342	DAPI	395	431	119	Cell Signaling Technology	4082 S	1:1000	0.03
TONSIL-2.4	10	3	PDL2	Alexa 488	FITC	485	525	71	Cell Signaling Technology	82723 S	1:200	0.5
TONSIL-2.4	11	3	A555 background		Cy3	555	590					0.5
TONSIL-2.4	12	3	p-cJun	Alexa 647	Cy5	640	690	116	Santa Cruz	sc-822	1:100	0.5
TONSIL-2.4	13	4	DAPI_4	Hoechst 33342	DAPI	395	431	119	Cell Signaling Technology	4082 S	1:1000	0.03
TONSIL-2.4	14	4	PDL2	Alexa 488	FITC	485	525	71	Cell Signaling Technology	82723 S	1:200	0.2
TONSIL-2.4	15	4	A555 background		Cy3	555	590					0.2
TONSIL-2.4	16	4	p-cJun	Alexa 647	Cy5	640	690	116	Santa Cruz	sc-822	1:100	0.2
TONSIL-2.4	17	5	DAPI_5	Hoechst 33342	DAPI	395	431	119	Cell Signaling Technology	4082 S	1:1000	0.03
TONSIL-2.4	18	5	GATA3	Alexa 488	FITC	485	525	35	BD Biosiences	560163	1:200	0.5
TONSIL-2.4	19	5	CD66b	Alexa 555	Cy3	555	590	43	Invitrogen	12-0666-41	1:300	0.5
TONSIL-2.4	20	5	CD14	Alexa 647	Cy5	640	690	83	Abcam	ab196169	1:1000	0.5
TONSIL-2.4	21	6	DAPI_6	Hoechst 33342	DAPI	395	431	119	Cell Signaling Technology	4082 S	1:1000	0.03
TONSIL-2.4	22	6	CD11b	Alexa 488	FITC	485	525	67	eBioscience	53-0196-80	1:200	0.5
TONSIL-2.4	23	6	CD68	Alexa 555	Cy3	555	590	1	Cell Signaling Technology	79594 S	1:1000	0.5
TONSIL-2.4	24	6	Her2	Alexa 647	Cy5	640	690	20	BioLegend	324412	1:200	0.5
TONSIL-2.4	25	7	DAPI_7	Hoechst 33342	DAPI	395	431	119	Cell Signaling Technology	4082 S	1:1000	0.03
TONSIL-2.4	26	7	PCNA	Alexa 488	FITC	485	525	72	Cell Signaling Technology	8580 S	1:1'1:6'1:70	0.2
TONSIL-2.4	27	7	CD133	Alexa 555	Cy3	555	590	45	eBioscience	12-1338-41	1:3'1:3'1:3	0.5
TONSIL-2.4	28	7	CD8a	Alexa 647	Cy5	640	690	62	eBioscience	50-0008-82	1:3'1:3'1:3	0.5
TONSIL-2.4	29	8	DAPI_8	Hoechst 33342	DAPI	395	431	119	Cell Signaling Technology	4082 S	1:1000	0.03
TONSIL-2.4	30	8	CD4	Alexa 488	FITC	485	525	108	R&D Systems	FAB8165G	1:300	0.5
TONSIL-2.4	31	8	CD31	Alexa 555	Cy3	555	590	107	R&D Systems	FAB3567P	1:100	0.5
TONSIL-2.4	32	8	CD103	Alexa 647	Cy5	640	690	24	BioLegend	350209	1:100	0.5
TONSIL-2.4	33	9	DAPI_9	Hoechst 33342	DAPI	395	431	119	Cell Signaling Technology	4082 S	1:1000	0.03
TONSIL-2.4	34	9	CD15	Alexa 488	FITC	485	525	4	BioLegend	301910	1:1'1:50	0.2
TONSIL-2.4	35	9	CD80	PE	Cy3	555	590	12	BioLegend	305207	1:1'1:50	0.5
TONSIL-2.4	36	9	CD20	Alexa 647	Cy5	640	690	64	eBioscience	50-0202-80	1:1000	0.2
TONSIL-2.4	37	10	DAPI_10	Hoechst 33342	DAPI	395	431	119	Cell Signaling Technology	4082 S	1:1000	0.03
TONSIL-2.4	38	10	CD11b	Alexa 488	FITC	485	525	94	Abcam	ab204271	1:300	0.5
TONSIL-2.4	39	10	Keratin	Alexa 555	Cy3	555	590	56	eBioscience	41-9003-82	1:1000	0.1
TONSIL-2.4	40	10	aSMA	Alexa 647	Cy5	640	690	65	eBioscience	50-9760-82	1:1000	0.2
TONSIL-2.4	41	11	DAPI_11	Hoechst 33342	DAPI	395	431	119	Cell Signaling Technology	4082 S	1:1000	0.03
TONSIL-2.4	42	11	TDP43	Alexa 488	FITC	485	525	80	Abcam	ab193842	1:1'1:50	0.5
TONSIL-2.4	43	11	FOXO1A	Alexa 555	Cy3	555	590	95	Abcam	ab207244	1:1'1:50	0.5
TONSIL-2.4	44	11	CD138	APC	Cy5	640	690	26	BioLegend	352308	1:1'1:50	0.5
TONSIL-2.4	45	12	DAPI_12	Hoechst 33342	DAPI	395	431	119	Cell Signaling Technology	4082 S	1:1000	0.03
TONSIL-2.4	46	12	IBA	Alexa 488	FITC	485	525	82	Abcam	ab195031	1:500	0.5
TONSIL-2.4	47	12	FoxP3	Alexa 555	Cy3	555	590	55	eBioscience	41-4777-82	1:1'1:50	0.5
TONSIL-2.4	48	12	CD16	Alexa 647	Cy5	640	690	114	Santa Cruz	sc-20052 AF647	1:200	0.5
TONSIL-2.5	1	1	DAPI_1	Hoechst 33342	DAPI	395	431	119	Cell Signaling Technology	4082 S	1:1000	0.03
TONSIL-2.5	2	1	A488 background		FITC	485	525					0.5
TONSIL-2.5	3	1	A555 background		Cy3	555	590					0.5
TONSIL-2.5	4	1	A647 background		Cy5	640	690					0.5
TONSIL-2.5	5	2	DAPI_2	Hoechst 33342	DAPI	395	431	119	Cell Signaling Technology	4082 S	1:1000	0.03
TONSIL-2.5	6	2	A488 background		FITC	485	525					0.2
TONSIL-2.5	7	2	A555 background		Cy3	555	590					0.2
TONSIL-2.5	8	2	A647 background		Cy5	640	690					0.2
TONSIL-2.5	9	3	DAPI_3	Hoechst 33342	DAPI	395	431	119	Cell Signaling Technology	4082 S	1:1000	0.03
TONSIL-2.5	10	3	CD2	Alexa 488	FITC	485	525	103	Abcam	ab37212	1:100	0.5
TONSIL-2.5	11	3	A555 background		Cy3	555	590					0.5
TONSIL-2.5	12	3	GATA3	Alexa 647	Cy5	640	690	110	Thermo-Fisher	MA1-028	1:100	0.5
TONSIL-2.5	13	4	DAPI_4	Hoechst 33342	DAPI	395	431	119	Cell Signaling Technology	4082 S	1:1000	0.03
TONSIL-2.5	14	4	CD2	Alexa 488	FITC	485	525	103	Abcam	ab37212	1:100	0.2
TONSIL-2.5	15	4	A555 background		Cy3	555	590					0.2
TONSIL-2.5	16	4	GATA3	Alexa 647	Cy5	640	690	110	Thermo-Fisher	MA1-028	1:100	0.2
TONSIL-2.5	17	5	DAPI_5	Hoechst 33342	DAPI	395	431	119	Cell Signaling Technology	4082 S	1:1000	0.03
TONSIL-2.5	18	5	IBA	Alexa 488	FITC	485	525	82	Abcam	ab195031	1:500	0.5
TONSIL-2.5	19	5	Keratin	Alexa 555	Cy3	555	590	56	eBioscience	41-9003-82	1:1000	0.5
TONSIL-2.5	20	5	PDL1	Alexa 647	Cy5	640	690	50	Cell Signaling Technology	15005 S	1:300	0.5
TONSIL-2.5	21	6	DAPI_6	Hoechst 33342	DAPI	395	431	119	Cell Signaling Technology	4082 S	1:1000	0.03
TONSIL-2.5	22	6	CD11a	Alexa 488	FITC	485	525	41	eBioscience	11-0119-41	1:200	0.5
TONSIL-2.5	23	6	CD3D	Alexa 555	Cy3	555	590	97	Abcam	ab208514	1:1'1:50	0.5
TONSIL-2.5	24	6	PD1	Alexa 647	Cy5	640	690	89	abcam	ab201825	1:200	0.5
TONSIL-2.5	25	7	DAPI_7	Hoechst 33342	DAPI	395	431	119	Cell Signaling Technology	4082 S	1:1000	0.03
TONSIL-2.5	26	7	GATA3	Alexa 488	FITC	485	525	35	BD Biosiences	560163	1:1'1:6'1:7	0.5
TONSIL-2.5	27	7	CD68	Alexa 555	Cy3	555	590	1	Cell Signaling Technology	79594 S	1:1'1:6'1:7	0.5
TONSIL-2.5	28	7	Beta-catenin	Alexa 647	Cy5	640	690	39	BioLegend	658705	1:500	0.5
TONSIL-2.5	29	8	DAPI_8	Hoechst 33342	DAPI	395	431	119	Cell Signaling Technology	4082 S	1:1000	0.03
TONSIL-2.5	30	8	PCNA	Alexa 488	FITC	485	525	72	Cell Signaling Technology	8580 S	1:1000	0.2
TONSIL-2.5	31	8	TIM1	Alexa 555	Cy3	555	590	27	BioLegend	353904	1:100	0.5
TONSIL-2.5	32	8	CD20	Alexa 647	Cy5	640	690	64	eBioscience	50-0202-80	1:1000	0.2
TONSIL-2.5	33	9	DAPI_9	Hoechst 33342	DAPI	395	431	119	Cell Signaling Technology	4082 S	1:1000	0.03
TONSIL-2.5	34	9	CD4	Alexa 488	FITC	485	525	108	R&D Systems	FAB8165G	1:300	0.5
TONSIL-2.5	35	9	aSMA	Alexa 555	Cy3	555	590	90	Abcam	ab202509	1:1000	0.1
TONSIL-2.5	36	9	CD45	Alexa 647	Cy5	640	690	10	BioLegend	304056	1:300	0.5
TONSIL-2.5	37	10	DAPI_10	Hoechst 33342	DAPI	395	431	119	Cell Signaling Technology	4082 S	1:1000	0.03
TONSIL-2.5	38	10	CD19	Alexa 488	FITC	485	525	84	Abcam	ab196468	1:200	0.5
TONSIL-2.5	39	10	pH2AX	Alexa 555	Cy3	555	590	36	BioLegend	613412	1:300	0.2
TONSIL-2.5	40	10	RunX3	Alexa 647	Cy5	640	690	66	eBioscience	50-9817-80	1:200	0.5
TONSIL-2.5	41	11	DAPI_11	Hoechst 33342	DAPI	395	431	119	Cell Signaling Technology	4082 S	1:1000	0.03
TONSIL-2.5	42	11	CD163	Alexa 488	FITC	485	525	101	Abcam	ab218293	1:300	0.5
TONSIL-2.5	43	11	CD66b	Alexa 555	Cy3	555	590	43	Thermo-Fisher	12-0666-41	1:100	0.5
TONSIL-2.5	44	11	Ki67	Alexa 647	Cy5	640	690	25	BioLegend	350509	1:200	0.5
TONSIL-2.5	45	12	DAPI_12	Hoechst 33342	DAPI	395	431	119	Cell Signaling Technology	4082 S	1:1000	0.03
TONSIL-2.5	46	12	LaminA/C	Alexa 488	FITC	485	525	73	Cell Signaling Technology	8617 S	1:300	0.5
TONSIL-2.5	47	12	NFATc1	Alexa 555	Cy3	555	590	38	BioLegend	649605	1:200	0.5
TONSIL-2.5	48	12	CD14	Alexa 647	Cy5	640	690	83	Abcam	ab196169	1:1000	0.5
TONSIL-2.6	1	1	DAPI_1	Hoechst 33342	DAPI	395	431	119	Cell Signaling Technology	4082 S	1:1000	0.03
TONSIL-2.6	2	1	A488 background		FITC	485	525					0.5
TONSIL-2.6	3	1	A555 background		Cy3	555	590					0.5
TONSIL-2.6	4	1	A647 background		Cy5	640	690					0.5
TONSIL-2.6	5	2	DAPI_2	Hoechst 33342	DAPI	395	431	119	Cell Signaling Technology	4082 S	1:1000	0.03
TONSIL-2.6	6	2	A488 background		FITC	485	525					0.2
TONSIL-2.6	7	2	A555 background		Cy3	555	590					0.2
TONSIL-2.6	8	2	A647 background		Cy5	640	690					0.2
TONSIL-2.6	9	3	DAPI_3	Hoechst 33342	DAPI	395	431	119	Cell Signaling Technology	4082 S	1:1000	0.03
TONSIL-2.6	10	3	Axl	Alexa 488	FITC	485	525	74	Cell Signaling Technology	8661 S	1:300	0.5
TONSIL-2.6	11	3	A555 background		Cy3	555	590					0.5
TONSIL-2.6	12	3	IDO	Alexa 647	Cy5	640	690	111	EMD-Millipore	MAB10009	1:100	0.5
TONSIL-2.6	13	4	DAPI_4	Hoechst 33342	DAPI	395	431	119	Cell Signaling Technology	4082 S	1:1000	0.03
TONSIL-2.6	14	4	Axl	Alexa 488	FITC	485	525	74	Cell Signaling Technology	8661 S	1:300	0.2
TONSIL-2.6	15	4	A555 background		Cy3	555	590					0.2
TONSIL-2.6	16	4	IDO	Alexa 647	Cy5	640	690	111	EMD-Millipore	MAB10009	1:100	0.2
TONSIL-2.6	17	5	DAPI_5	Hoechst 33342	DAPI	395	431	119	Cell Signaling Technology	4082 S	1:1000	0.03
TONSIL-2.6	18	5	TIM3	Alexa 488	FITC	485	525	69	Cell Signaling Technology	54669 S	1:200	0.5
TONSIL-2.6	19	5	Her2	Alexa 555	Cy3	555	590	57	eBioscience	41-9757-80	1:300	0.5
TONSIL-2.6	20	5	CD8a	Alexa 647	Cy5	640	690	62	eBioscience	50-0008-82	1:200	0.5
TONSIL-2.6	21	6	DAPI_6	Hoechst 33342	DAPI	395	431	119	Cell Signaling Technology	4082 S	1:1000	0.03
TONSIL-2.6	22	6	CD123	Alexa 488	FITC	485	525	16	BioLegend	306035	1:200	0.5
TONSIL-2.6	23	6	FoxP3	Alexa 555	Cy3	555	590	55	eBioscience	41-4777-82	1:1'1:50	0.5
TONSIL-2.6	24	6	CD20	Alexa 647	Cy5	640	690	64	eBioscience	50-0202-80	1:1000	0.2
TONSIL-2.6	25	7	DAPI_7	Hoechst 33342	DAPI	395	431	119	Cell Signaling Technology	4082 S	1:1000	0.03
TONSIL-2.6	26	7	CD163	Alexa 488	FITC	485	525	101	Abcam	ab218293	1:500	0.5
TONSIL-2.6	27	7	NFATc1	Alexa 555	Cy3	555	590	38	BioLegend	649605	1:3'1:3'1:3	0.5
TONSIL-2.6	28	7	ABCC1	Alexa 647	Cy5	640	690	32	BioLegend	370203	1:500	0.5
TONSIL-2.6	29	8	DAPI_8	Hoechst 33342	DAPI	395	431	119	Cell Signaling Technology	4082 S	1:1000	0.03
TONSIL-2.6	30	8	CD11b	Alexa 488	FITC	485	525	94	Abcam	ab204271	1:300	0.5
TONSIL-2.6	31	8	CD3D	Alexa 555	Cy3	555	590	97	Abcam	ab208514	1:1'1:50	0.5
TONSIL-2.6	32	8	IBA1	Alexa 647	Cy5	640	690	104	Bioss	AIF1	1:100	0.5
TONSIL-2.6	33	9	DAPI_9	Hoechst 33342	DAPI	395	431	119	Cell Signaling Technology	4082 S	1:1000	0.03
TONSIL-2.6	34	9	CD11b	Alexa 488	FITC	485	525	67	eBioscience	53-0196-80	1:200	0.5
TONSIL-2.6	35	9	CD68	Alexa 555	Cy3	555	590	1	Cell Signaling Technology	79594 S	1:1000	0.5
TONSIL-2.6	36	9	CD206	Alexa 647	Cy5	640	690	18	BioLegend	321116	1:1'1:50	0.5
TONSIL-2.6	37	10	DAPI_10	Hoechst 33342	DAPI	395	431	119	Cell Signaling Technology	4082 S	1:1000	0.03
TONSIL-2.6	38	10	IBA	Alexa 488	FITC	485	525	82	Abcam	ab195031	1:500	0.5
TONSIL-2.6	39	10	pH3	Alexa 555	Cy3	555	590	52	Cell Signaling Technology	3475 S	1:1000	0.1
TONSIL-2.6	40	10	Ki67	Alexa 647	Cy5	640	690	25	BioLegend	350509	1:1000	0.5
TONSIL-2.6	41	11	DAPI_11	Hoechst 33342	DAPI	395	431	119	Cell Signaling Technology	4082 S	1:1000	0.03
TONSIL-2.6	42	11	GATA3	Alexa 488	FITC	485	525	35	BD Biosiences	560163	1:100	0.5
TONSIL-2.6	43	11	VEGFR2	PE	Cy3	555	590	46	Cell Signaling Technology	12634 S	1:1'1:50	0.2
TONSIL-2.6	44	11	IRF1	Alexa 647	Cy5	640	690	49	Cell Signaling Technology	14105 S	1:200	0.5
TONSIL-2.6	45	12	DAPI_12	Hoechst 33342	DAPI	395	431	119	Cell Signaling Technology	4082 S	1:1000	0.03
TONSIL-2.6	46	12	PCNA	Alexa 488	FITC	485	525	72	Cell Signaling Technology	8580 S	1:1000	0.2
TONSIL-2.6	47	12	CD11c	Alexa 555	Cy3	555	590	58	eBioscience	41-9761-80	1:200	0.5
TONSIL-2.6	48	12	CD45	Alexa 647	Cy5	640	690	10	BioLegend	304056	1:300	0.5
TONSIL-2.7	1	1	DAPI_1	Hoechst 33342	DAPI	395	431	119	Cell Signaling Technology	4082 S	1:1000	0.03
TONSIL-2.7	2	1	A488 background		FITC	485	525					0.5
TONSIL-2.7	3	1	A555 background		Cy3	555	590					0.5
TONSIL-2.7	4	1	A647 background		Cy5	640	690					0.5
TONSIL-2.7	5	2	DAPI_2	Hoechst 33342	DAPI	395	431	119	Cell Signaling Technology	4082 S	1:1000	0.03
TONSIL-2.7	6	2	A488 background		FITC	485	525					0.2
TONSIL-2.7	7	2	A555 background		Cy3	555	590					0.2
TONSIL-2.7	8	2	A647 background		Cy5	640	690					0.2
TONSIL-2.7	9	3	DAPI_3	Hoechst 33342	DAPI	395	431	119	Cell Signaling Technology	4082 S	1:1000	0.03
TONSIL-2.7	10	3	STING	Alexa 488	FITC	485	525	48	Cell Signaling Technology	13647 S	1:100	0.5
TONSIL-2.7	11	3	A555 background		Cy3	555	590					0.5
TONSIL-2.7	12	3	CD1c	Alexa 647	Cy5	640	690	75	Abcam	ab156708	1:200	0.5
TONSIL-2.7	13	4	DAPI_4	Hoechst 33342	DAPI	395	431	119	Cell Signaling Technology	4082 S	1:1000	0.03
TONSIL-2.7	14	4	STING	Alexa 488	FITC	485	525	48	Cell Signaling Technology	13647 S	1:100	0.2
TONSIL-2.7	15	4	A555 background		Cy3	555	590					0.2
TONSIL-2.7	16	4	CD1c	Alexa 647	Cy5	640	690	75	Abcam	ab156708	1:200	0.2
TONSIL-2.7	17	5	DAPI_5	Hoechst 33342	DAPI	395	431	119	Cell Signaling Technology	4082 S	1:1000	0.03
TONSIL-2.7	18	5	CD11b	Alexa 488	FITC	485	525	67	eBioscience	53-0196-80	1:300	0.5
TONSIL-2.7	19	5	CD45R	Alexa 555	Cy3	555	590	53	invitrogen	41-0452-80	1:300	0.5
TONSIL-2.7	20	5	CD25	Alexa 647	Cy5	640	690	7	BioLegend	302617	1:1'1:50	0.5
TONSIL-2.7	21	6	DAPI_6	Hoechst 33342	DAPI	395	431	119	Cell Signaling Technology	4082 S	1:1000	0.03
TONSIL-2.7	22	6	CD163	Alexa 488	FITC	485	525	101	Abcam	ab218293	1:300	0.5
TONSIL-2.7	23	6	CD1c	Alexa 555	Cy3	555	590	21	BioLegend	331505	1:200	0.5
TONSIL-2.7	24	6	CD8a	Alexa 647	Cy5	640	690	62	eBioscience	50-0008-82	1:200	0.5
TONSIL-2.7	25	7	DAPI_7	Hoechst 33342	DAPI	395	431	119	Cell Signaling Technology	4082 S	1:1000	0.03
TONSIL-2.7	26	7	IBA	Alexa 488	FITC	485	525	82	Abcam	ab195031	1:8'1:3'1:3	0.5
TONSIL-2.7	27	7	Keratin	Alexa 555	Cy3	555	590	56	eBioscience	41-9003-82	1:1'1:6'1:70	0.1
TONSIL-2.7	28	7	CD45	Alexa 647	Cy5	640	690	10	BioLegend	304056	1:500	0.5
TONSIL-2.7	29	8	DAPI_8	Hoechst 33342	DAPI	395	431	119	Cell Signaling Technology	4082 S	1:1000	0.03
TONSIL-2.7	30	8	BRD7	Alexa 488	FITC	485	525	105	Aviva	ARP39018-P050	1:100	0.5
TONSIL-2.7	31	8	Beta-Tubulin	Alexa 555	Cy3	555	590	51	Cell Signaling Technology	2116 S	1:300	0.5
TONSIL-2.7	32	8	CD14	Alexa 647	Cy5	640	690	83	Abcam	ab196169	1:1000	0.5
TONSIL-2.7	33	9	DAPI_9	Hoechst 33342	DAPI	395	431	119	Cell Signaling Technology	4082 S	1:1000	0.03
TONSIL-2.7	34	9	CD16	Alexa 488	FITC	485	525	5	BioLegend	302019	1:1'1:50	0.5
TONSIL-2.7	35	9	FoxP3	Alexa 555	Cy3	555	590	55	eBioscience	41-4777-82	1:1'1:50	0.5
TONSIL-2.7	36	9	CD134	Alexa 647	Cy5	640	690	23	BioLegend	350018	1:1'1:50	0.5
TONSIL-2.7	37	10	DAPI_10	Hoechst 33342	DAPI	395	431	119	Cell Signaling Technology	4082 S	1:1000	0.03
TONSIL-2.7	38	10	CD4	Alexa 488	FITC	485	525	108	R&D Systems	FAB8165G	1:300	0.5
TONSIL-2.7	39	10	CD11c	Alexa 555	Cy3	555	590	58	eBioscience	41-9761-80	1:200	0.5
TONSIL-2.7	40	10	CD20	Alexa 647	Cy5	640	690	64	eBioscience	50-0202-80	1:1000	0.2
TONSIL-2.7	41	11	DAPI_11	Hoechst 33342	DAPI	395	431	119	Cell Signaling Technology	4082 S	1:1000	0.03
TONSIL-2.7	42	11	Fibronectin	Alexa 488	FITC	485	525	85	Abcam	ab198933	1:1000	0.5
TONSIL-2.7	43	11	pAur	Alexa 555	Cy3	555	590	47	Cell Signaling Technology	13464 S	1:200	0.5
TONSIL-2.7	44	11	STING	Alexa 647	Cy5	640	690	86	Abcam	ab198952	1:1'1:50	0.2
TONSIL-2.7	45	12	DAPI_12	Hoechst 33342	DAPI	395	431	119	Cell Signaling Technology	4082 S	1:1000	0.03
TONSIL-2.7	46	12	CD11b	Alexa 488	FITC	485	525	94	Abcam	ab204271	1:300	0.5
TONSIL-2.7	47	12	CD3D	Alexa 555	Cy3	555	590	97	Abcam	ab208514	1:1'1:50	0.5
TONSIL-2.7	48	12	PD1	Alexa 647	Cy5	640	690	89	Abcam	ab201825	1:200	0.5
TONSIL-2.8	1	1	DAPI_1	Hoechst 33342	DAPI	395	431	119	Cell Signaling Technology	4082 S	1:1000	0.03
TONSIL-2.8	2	1	A488 background		FITC	485	525					0.5
TONSIL-2.8	3	1	A555 background		Cy3	555	590					0.5
TONSIL-2.8	4	1	A647 background		Cy5	640	690					0.5
TONSIL-2.8	5	2	DAPI_2	Hoechst 33342	DAPI	395	431	119	Cell Signaling Technology	4082 S	1:1000	0.03
TONSIL-2.8	6	2	A488 background		FITC	485	525					0.2
TONSIL-2.8	7	2	A555 background		Cy3	555	590					0.2
TONSIL-2.8	8	2	A647 background		Cy5	640	690					0.2
TONSIL-2.8	9	3	DAPI_3	Hoechst 33342	DAPI	395	431	119	Cell Signaling Technology	4082 S	1:1000	0.03
TONSIL-2.8	10	3	CD69	Alexa 488	FITC	485	525	92	Abcam	ab202909	1:100	0.5
TONSIL-2.8	11	3	A555 background		Cy3	555	590					0.5
TONSIL-2.8	12	3	RORyT	Alexa 647	Cy5	640	690	112	EMD-Millipore	MABF81	1:50	0.5
TONSIL-2.8	13	4	DAPI_4	Hoechst 33342	DAPI	395	431	119	Cell Signaling Technology	4082 S	1:1000	0.03
TONSIL-2.8	14	4	CD69	Alexa 488	FITC	485	525	92	Abcam	ab202909	1:100	0.2
TONSIL-2.8	15	4	A555 background		Cy3	555	590					0.2
TONSIL-2.8	16	4	RORyT	Alexa 647	Cy5	640	690	112	EMD-Millipore	MABF81	1:50	0.2
TONSIL-2.8	17	5	DAPI_5	Hoechst 33342	DAPI	395	431	119	Cell Signaling Technology	4082 S	1:1000	0.03
TONSIL-2.8	18	5	CD163	Alexa 488	FITC	485	525	101	Abcam	ab218293	1:300	0.5
TONSIL-2.8	19	5	CD11c	Alexa 555	Cy3	555	590	58	eBioscience	41-9761-80	1:200	0.5
TONSIL-2.8	20	5	CD45	Alexa 647	Cy5	640	690	10	BioLegend	304056	1:300	0.5
TONSIL-2.8	21	6	DAPI_6	Hoechst 33342	DAPI	395	431	119	Cell Signaling Technology	4082 S	1:1000	0.03
TONSIL-2.8	22	6	IBA	Alexa 488	FITC	485	525	82	Abcam	ab195031	1:500	0.5
TONSIL-2.8	23	6	CD11a	Alexa 555	Cy3	555	590	3	BioLegend	301207	1:200	0.5
TONSIL-2.8	24	6	CD3	Alexa 647	Cy5	640	690	2	BioLegend	300422	1:200	0.5
TONSIL-2.8	25	7	DAPI_7	Hoechst 33342	DAPI	395	431	119	Cell Signaling Technology	4082 S	1:1000	0.03
TONSIL-2.8	26	7	TIM3	Alexa 488	FITC	485	525	69	Cell Signaling Technology	54669 S	1:3'1:3'1:3	0.5
TONSIL-2.8	27	7	CD3D	Alexa 555	Cy3	555	590	97	Abcam	ab208514	1:2'1:50	0.5
TONSIL-2.8	28	7	PDL1	Alexa 647	Cy5	640	690	50	Cell Signaling Technology	15005 S	1:500	0.5
TONSIL-2.8	29	8	DAPI_8	Hoechst 33342	DAPI	395	431	119	Cell Signaling Technology	4082 S	1:1000	0.03
TONSIL-2.8	30	8	GATA3	Alexa 488	FITC	485	525	35	BD Biosiences	560163	1:100	0.5
TONSIL-2.8	31	8	FoxP3	Alexa 555	Cy3	555	590	55	eBioscience	41-4777-82	1:1'1:50	0.5
TONSIL-2.8	32	8	Annexin V	Alexa 647	Cy5	640	690	37	BioLegend	640911	1:1'1:50	0.5
TONSIL-2.8	33	9	DAPI_9	Hoechst 33342	DAPI	395	431	119	Cell Signaling Technology	4082 S	1:1000	0.03
TONSIL-2.8	34	9	PCNA	Alexa 488	FITC	485	525	72	Cell Signaling Technology	8580 S	1:1000	0.2
TONSIL-2.8	35	9	Keratin	Alexa 555	Cy3	555	590	56	eBioscience	41-9003-82	1:1000	0.1
TONSIL-2.8	36	9	CD14	Alexa 647	Cy5	640	690	83	Abcam	ab196169	1:1000	0.5
TONSIL-2.8	37	10	DAPI_10	Hoechst 33342	DAPI	395	431	119	Cell Signaling Technology	4082 S	1:1000	0.03
TONSIL-2.8	38	10	CD38	Alexa 488	FITC	485	525	9	BioLegend	303511	1:1'1:50	0.5
TONSIL-2.8	39	10	CD68	Alexa 555	Cy3	555	590	1	Cell Signaling Technology	79594 S	1:1000	0.5
TONSIL-2.8	40	10	CD8a	Alexa 647	Cy5	640	690	114	eBioscience	50-0008-82	1:200	0.5
TONSIL-2.8	41	11	DAPI_11	Hoechst 33342	DAPI	395	431	119	Cell Signaling Technology	4082 S	1:1000	0.03
TONSIL-2.8	42	11	S100a	Alexa 488	FITC	485	525	96	Abcam	ab207367	1:1'1:50	0.1
TONSIL-2.8	43	11	SQSTM1	Alexa 555	Cy3	555	590	93	Abcam	ab203430	1:1'1:50	0.5
TONSIL-2.8	44	11	Ki67	Alexa 647	Cy5	640	690	44	Cell Signaling Technology	12075 S	1:300	0.05
TONSIL-2.8	45	12	DAPI_12	Hoechst 33342	DAPI	395	431	119	Cell Signaling Technology	4082 S	1:1000	0.03
TONSIL-2.8	46	12	EGFR	Alexa 488	FITC	485	525	70	Cell Signaling Technology	5616 S	1:500	0.5
TONSIL-2.8	47	12	aSMA	Alexa 555	Cy3	555	590	90	Abcam	ab202509	1:1000	0.1
TONSIL-2.8	48	12	CD20	Alexa 647	Cy5	640	690	64	eBioscience	50-0202-80	1:1000	0.2

**Online-only Table 4:** Description of Features.

Feature	Description
FieldID	Split 6000 × 6000 field cells were segmented from
CellId	Unique cell identifier in a specific image
DAPI1	Log-transformed mean intensity of pixels covered by segmentation for specified marker
A488background1	Log-transformed mean intensity of pixels covered by segmentation for specified marker
A555background1	Log-transformed mean intensity of pixels covered by segmentation for specified marker
A647background1	Log-transformed mean intensity of pixels covered by segmentation for specified marker
DAPI2	Log-transformed mean intensity of pixels covered by segmentation for specified marker
A488background2	Log-transformed mean intensity of pixels covered by segmentation for specified marker
A555background2	Log-transformed mean intensity of pixels covered by segmentation for specified marker
A647background2	Log-transformed mean intensity of pixels covered by segmentation for specified marker
DAPI3	Log-transformed mean intensity of pixels covered by segmentation for specified marker
A488background3	Log-transformed mean intensity of pixels covered by segmentation for specified marker
LAG3	Log-transformed mean intensity of pixels covered by segmentation for specified marker
ARL13B	Log-transformed mean intensity of pixels covered by segmentation for specified marker
DAPI4	Log-transformed mean intensity of pixels covered by segmentation for specified marker
KI67	Log-transformed mean intensity of pixels covered by segmentation for specified marker
KERATIN	Log-transformed mean intensity of pixels covered by segmentation for specified marker
PD1	Log-transformed mean intensity of pixels covered by segmentation for specified marker
DAPI5	Log-transformed mean intensity of pixels covered by segmentation for specified marker
CD45RB	Log-transformed mean intensity of pixels covered by segmentation for specified marker
CD3D	Log-transformed mean intensity of pixels covered by segmentation for specified marker
PDL1	Log-transformed mean intensity of pixels covered by segmentation for specified marker
DAPI6	Log-transformed mean intensity of pixels covered by segmentation for specified marker
CD4	Log-transformed mean intensity of pixels covered by segmentation for specified marker
CD45	Log-transformed mean intensity of pixels covered by segmentation for specified marker
CD8A	Log-transformed mean intensity of pixels covered by segmentation for specified marker
DAPI7	Log-transformed mean intensity of pixels covered by segmentation for specified marker
CD163	Log-transformed mean intensity of pixels covered by segmentation for specified marker
CD68	Log-transformed mean intensity of pixels covered by segmentation for specified marker
CD14	Log-transformed mean intensity of pixels covered by segmentation for specified marker
DAPI8	Log-transformed mean intensity of pixels covered by segmentation for specified marker
CD11B	Log-transformed mean intensity of pixels covered by segmentation for specified marker
FOXP3	Log-transformed mean intensity of pixels covered by segmentation for specified marker
CD21	Log-transformed mean intensity of pixels covered by segmentation for specified marker
DAPI9	Log-transformed mean intensity of pixels covered by segmentation for specified marker
IBA1	Log-transformed mean intensity of pixels covered by segmentation for specified marker
ASMA	Log-transformed mean intensity of pixels covered by segmentation for specified marker
CD20	Log-transformed mean intensity of pixels covered by segmentation for specified marker
DAPI10	Log-transformed mean intensity of pixels covered by segmentation for specified marker
CD19	Log-transformed mean intensity of pixels covered by segmentation for specified marker
GFAP	Log-transformed mean intensity of pixels covered by segmentation for specified marker
GTUBULIN	Log-transformed mean intensity of pixels covered by segmentation for specified marker
DAPI11	Log-transformed mean intensity of pixels covered by segmentation for specified marker
LAMINAC	Log-transformed mean intensity of pixels covered by segmentation for specified marker
BANF1	Log-transformed mean intensity of pixels covered by segmentation for specified marker
LAMINB	Log-transformed mean intensity of pixels covered by segmentation for specified marker
Area	Matlab regionprops function: “Actual number of pixels in the region, returned as a scalar.”
Eccentricity	Matlab regionprops function: “Eccentricity of the ellipse that has the same second-moments as the region, returned as a scalar. The eccentricity is the ratio of the distance between the foci of the ellipse and its major axis length. The value is between 0 and 1. (0 and 1 are degenerate cases. An ellipse whose eccentricity is 0 is actually a circle, while an ellipse whose eccentricity is 1 is a line segment.)”
Solidity	Matlab regionprops function: “Proportion of the pixels in the convex hull that are also in the region, returned as a scalar. Computed as Area/ConvexArea.”
Extent	Matlab regionprops function: “Ratio of pixels in the region to pixels in the total bounding box, returned as a scalar. Computed as the Area divided by the area of the bounding box.”
EulerNumber	Matlab regionprops function: “Number of objects in the region minus the number of holes in those objects, returned as a scalar. This property is supported only for 2-D label matrices. regionprops uses 8-connectivity to compute the Euler number measurement.”
Perimeter	Matlab regionprops function: “Distance around the boundary of the region returned as a scalar. regionprops computes the perimeter by calculating the distance between each adjoining pair of pixels around the border of the region.”
MajorAxisLength	Matlab regionprops function: “Length (in pixels) of the major axis of the ellipse that has the same normalized second central moments as the region, returned as a scalar.”
MinorAxisLength	Matlab regionprops function: “Length (in pixels) of the minor axis of the ellipse that has the same normalized second central moments as the region, returned as a scalar.”
Orientation	Matlab regionprops function: “Angle between the x-axis and the major axis of the ellipse that has the same second-moments as the region, returned as a scalar. The value is in degrees, ranging from −90 degrees to 90 degrees.”
X_position	X-position of the centroid calculated as the center of mass of the cell.
Y_position	Y-position of the centroid calculated as the center of mass of the cell.
Percent_Touching	CellProfiler2.0 function: “Percent of the object’s boundary pixels that touch neighbors, after the objects have been expanded to the specified distance.”
Number_Neighbors	Number of neighboring cells in a specified pixel extension.
neighbor_*	Cell identifier for all neighboring cells in a specified pixel extension.

**Online-only Table 5:** Script Annotation.

Script File Name	Programming Language	Description
imagej_basic_ashlar.py	Python	Script used to run BaSiC and ASHLAR algorithms.
run_ashlar_csv_batch.py	Python	Script used to run imagej_basic_ashlar.py for multiple samples in a batch
ome_to_individual_tiff.m	MATLAB	Script used to extract, rename, and save the highest resolution TIFF images for each marker from the OME-TIFF file
split_ome_tiff.m	MATLAB	Script used to generate 6000 × 6000 pixel split regions and 250 × 250 pixel cropped regions from ome.tiff file
convert_slices_from_Z_to_X.ijm	ImageJ	Script used to convert image slices of cropped regions and training regions from Z to X for compatibility with ilastik
segment_from_ilastik_LUNG-1-LN.m	MATLAB	Script used to generate segmentation masks from ilastik probability masks for LUNG-1-LN
segment_from_ilastik_LUNG-2-BR.m	MATLAB	Script used to generate segmentation masks from ilastik probability masks for LUNG-2-BR
segment_from_ilastik_LUNG-3-PR.m	MATLAB	Script used to generate segmentation masks from ilastik probability masks for LUNG-3-PR
segment_from_ilastik_TONSIL-1.m	MATLAB	Script used to generate segmentation masks from ilastik probability masks for TONSIL-1
combined_to_master_table	MATLAB	Script used to convert histoCAT tables from each split region into a master table for each sample
